# Comparative transcriptomic analysis of the different developmental stages of ovary in red swamp crayfish *Procambarus clarkii*

**DOI:** 10.1186/s12864-021-07537-x

**Published:** 2021-03-21

**Authors:** Yizhi Zhong, Wenbin Zhao, Zhangsheng Tang, Liming Huang, Xiangxing Zhu, Xiang Liang, Aifen Yan, Zhifa Lu, Yanling Yu, Dongsheng Tang, Dapeng Wang, Zhuanling Lu

**Affiliations:** 1 Guangxi Academy of Fishery Sciences/Guangxi Key Laboratory of Aquatic Genetic Breeding and Healthy Aquaculture, Nanning, 530021 China; 2 Guangdong Provincial Key Laboratory of Animal Molecular Design and Precise Breeding, Guangdong Provincial Engineering and Technology Research Center for Gene Editing, School of Medical Engineering, Foshan University, Foshan, 528225 China; 3 Development Research Institute of Agro-animal Husbandry Industry, Guangxi University, Nanning, 530004 China

**Keywords:** Different developmental stages, Molecular mechanisms, Ovary, *Procambarus clarkii*, Transcriptomics

## Abstract

**Background:**

The red swamp crayfish *Procambarus clarkii* is a freshwater species that possesses high adaptability, environmental tolerance, and fecundity. *P. clarkii* is artificially farmed on a large scale in China. However, the molecular mechanisms of ovarian development in *P. clarkii* remain largely unknown. In this study, we identified four stages of *P. clarkii* ovary development, the previtellogenic stage (stage I), early vitellogenic stage (stage II), middle vitellogenic stage (stage III), and mature stage (stage IV) and compared the transcriptomics among these four stages through next-generation sequencing (NGS).

**Results:**

The total numbers of clean reads of the four stages ranged from 42,013,648 to 62,220,956. A total of 216,444 unigenes were obtained, and the GC content of most unigenes was slightly less than the AT content. Principal Component Analysis (PCA) and Anosim analysis demonstrated that the grouping of these four stages was feasible, and each stage could be distinguished from the others. In the expression pattern analysis, 2301 genes were continuously increase from stage I to stage IV, and 2660 genes were sharply decrease at stage IV compared to stages I-III. By comparing each of the stages at the same time, four clusters of differentially expressed genes (DEGs) were found to be uniquely highly expressed in stage I (136 genes), stage II (43 genes), stage III-IV (49 genes), and stage IV (22 genes), thus exhibiting developmental stage specificity. Moreover, in comparisons between adjacent stages, the number of DEGs between stage III and IV was the highest. GO enrichment analysis demonstrated that nutrient reservoir activity was highest at stage II and that this played a foreshadowing role in ovarian development, and the GO terms of cell, intracellular and organelle participated in the ovary maturation during later stages. In addition, KEGG pathway analysis revealed that the early development of the ovary was mainly associated with the PI3K-Akt signaling pathway and focal adhesion; the middle developmental period was related to apoptosis, lysine biosynthesis, and the NF-kappa B signaling pathway; the late developmental period was involved with the cell cycle and the p53 signaling pathway.

**Conclusion:**

These transcriptomic data provide insights into the molecular mechanisms of ovarian development in *P. clarkii*. The results will be helpful for improving the reproduction and development of this aquatic species.

**Supplementary Information:**

The online version contains supplementary material available at 10.1186/s12864-021-07537-x.

## Background

The red swamp crayfish *Procambarus clarkii* originated in south-central America and northeastern Mexico [1]. The freshwater crayfish is an invasive species now widely distributed in Europe, Africa, and Asia [2–5]. *P. clarkii* was first introduced into Nanjing, China, from Japan in 1929 [6], and at present it can be found in freshwater habitats such as rivers, swamps, sloughs, and paddy fields [5]. Although *P. clarkii* could lead to economic losses and declines biodiversity [7], the crayfish is one of the most important aquaculture resources [7–9], since it is welcomed by a vast number of consumers for its delicious taste and high meat quality. As a successful invasive species, *P. clarkii* has advantageous traits including a short life cycle, high fecundity, and high disease resistance [5, 6]. The species is highly adaptable and can disperse widely in the habitat and tolerate diverse environmental conditions [4, 10]. Furthermore, *P. clarkii* has retained high levels of genetic diversity in both wild populations [5, 6, 9, 11, 12] and commercial populations [13]; this contributes to avoiding the harmful effects of inbreeding, for adapting to different environments [14], and in the selection of good breeding germplasm for crayfish artificial culture [5]. At present, *P. clarkii* farming has become an important industry in China, with production reaching 1,638,700 tons and a total output value of 369 billion China Yuan (CNY) in 2018 [15]. Some successful reproductive results for commercial *P. clarkii* have been reported from areas such as Qianjiang, Hubei province, where breeding grounds of *P. clarkii* in China, extend over 200 ha and enclose a variety of artificial ponds [16]. The red swamp crayfish-rice culture is the major model for production of *P. clarkii* in China, a farming scheme that not only makes significant improvements in rural livelihood and food security but also contributes to eco-environmental benefits and sustainable development [17].

As a model organism, *P. clarkii* is not only used to investigate invasive routes and dispersal patterns [11, 18], but also to perform research on animal behavior [19–21] and environmental stress and toxicity [22–25]. With the rapid development of the aquaculture industry, *P. clarkii* is often infected by various pathogens such as bacteria, viruses, and spiroplasmas [26–29], resulting in severe decreases in *P. clarkii* production. Therefore, significant anti-microbial research and studies of the immune response of *P. clarkii* have been performed, especially using transcriptome analysis by next-generation sequencing (NGS) [29–32]. NGS is a high-throughput sequencing technology and constitutes a variety of strategies that depend on a combination of template preparation, sequencing and imaging, and genome alignment and assembly methods [33, 34]. Compared with the traditional Sanger sequencing technology, the NGS can produce an enormous volume of sequence data at an unprecedented level of sensitivity and accuracy, in shorter times, and at a much cheaper cost [33, 35]. Combined with the de novo assembly methods such as Trinity, the full-length transcriptome assembly from NGS data can be implemented without a reference genome and does not require the correct alignment of reads to known splice sites [35, 36]. Currently, NGS is frequently used to analyze the transcriptome variation of *P. clarkii* in a variety of research areas, including pathogen infection as mentioned above [26–29], the immune system [31, 37], neurohormone regulation [38], and gonadal development [37, 39].The NGS transcriptomic analysis of *P. clarkii* revealed that the ovary and testis, the major reproductive organs, were more closely related to the pathways of DNA replication, cell cycle, and meiosis-yeast compared to other non-reproductive organs (e.g., hepatopancreas and muscle) [37]. In addition, using 454 pyrosequencing technology, a differential expression analysis between the sexually mature ovary and testis of *P. clarkii* was performed, and the results identified gonadal development related genes that were highly expressed in ovary and testis [39]. However, we still have a limited understanding of the gonadal development of *P. clarkii*. In order to promote the development of the *P clarkii* industry and build a comprehensive breeding system, the developmental mechanisms of male and female *P. clarkii* should be further investigated.

In female *P. clarkii*, oocyte development is classified into seven stages according to morphological characteristics. These are oogonial, immature, avitellogenic, early vitellogenic, midvitellogenic, late vitellogenic, and postvitellogenic-resorptive stages. Except for the oogonial stage, the remaining stages could occur mature oocytes [40, 41]. Ovarian maturation includes an increase in size as the oocytes proliferate and increase in diameter during yolk and lipovitellin uptake [40, 42]. Hence, based on the size and color of the ovary, ovarian development of *P. clarkii* can be separated into five stages in the order of non-developed ovary (transparent), undifferentiated ovary (white), poorly developed ovary (yellow), developed ovary (orange), and mature ovary (brown) [41, 43]. To date, some researchers have investigated factors that impact ovarian maturation in *P. clarkii*, including chemical compounds, steroids, and herbicides. Treatment of methylfarnesoate (MF) for different time periods could stimulate and enhance the ovarian maturation of *P. clarkii* [42], and MF alone or in combination with 17β-estradiol (but not in combination with JHIII (juvenile hormone III) or 17α-hydroxyprogesterone) could improve oocyte growth through stimulating the synthesis of vitellin in the ovary [44]. However, 17α-hydroxyprogesterone could significantly increase the gonadosomatic index (GSI) and directly stimulate vitellogenin production in *P. clarkii* [45], indicating that 17α-hydroxyprogesterone was in competition with MF in the ovary or that it was involved in a negative feedback loop [44]. Interestingly, the ovary was the main target organ for selenium (Se) accumulation, and an appropriate concentration of Se in the diet could remarkably improve the spawning rate and promote synchronized ovulation of *P. clarkii* [46]. Moreover, Atrazine, a widely used herbicide, could reduce vitellogenin content in the ovary and decrease the oocyte size in *P. clarkii* [47], so the crayfish-rice culture system should consider the effect of Atrazine on the reproductive performance of the crayfish.

At present, the proteomic comparison between previtellogenic and vitellogenic ovaries of *P. clarkii* is performed using two-dimensional gel electrophoresis and mass spectrometry [48], but the information obtained has been sparse, with only 22 differentially expressed proteins being identified. More recently, the transcriptome information from ovaries at stage IV of *P. clarkii* has expanded our understanding of ovarian development and embryogenesis and demonstrated that pcRDH11 may play an essential role in this aspect [49]. However, the molecular mechanisms of the ovarian developmental process in *P. clarkii* remain poorly understood, and this hinders our understanding of reproduction and thereby affects the artificial breeding industry of *P. clarkii*. Herein, we selected the final four stages of ovarian development of *P. clarkii* from the five stages reported in the previous studies [41, 43], the previtellogenic stage (undifferentiated ovary, stage I), the early vitellogenic stage (poorly developed ovary, stage II), the middle vitellogenic stage (developed ovary, stage III), and the mature stage (mature ovary, stage IV) to perform transcriptome comparisons between different stages through NGS. The results will provide insights into the molecular mechanisms of ovarian development of *P. clarkii*.

## Materials and methods

### Ovarian tissue collection and identification of different developmental stages

The *P. clarkii* used in this study were cultured at a commercial farm in Laibin (23°39′92′′N, 109°23′34′′E), Guangxi, China, that has adopted the crayfish-rice culture pattern. Crayfish were captured monthly from May to September, 2019 using cylindrical traps, and the female crayfish were transferred to water tanks with adequate aeration at 28 °C for three days. The ovaries were collected and photographed to record their morphology and color, then immediately immersed in RNA preservation buffer (#R0118, Beyotime, China), frozen in liquid nitrogen, and stored at − 80 °C until use.

The development of the ovary was separated into four stages according to the morphology and color, as the previtellogenic stage (stage I, yellowish white), the early vitellogenic stage (stage II, yellow), the middle vitellogenic stage (stage III, dark orange or light brown), and the mature stage (stage IV, dark brown or black). To confirm the classification by histology, a portion of each ovary tissues was selected for HE staining. Finally, three samples of each stage were identified, defined as stage I (I_7, I_19 and I_20), stage II (II_17, II_27 and II_30), stage III (III_33, III_49 and III_52), and stage IV (IV_35, IV_36 and IV_37).

### RNA extraction, cDNA library construction, and Illumina sequencing

The total RNA of each ovary sample was extracted using Total RNA Extractor (Trizol) (B511311, Sangon, Shanghai, China) according to the manufacturer’s instructions. A Qubit RNA HS Assay Kit (Q32855, Invitrogen, Carlsbad, CA, USA) was used to detect the sample RNA concentration using a Qubit Fluorometer (Q32866, Invitrogen, Carlsbad, CA, USA). Agarose gel electrophoresis was used to detect RNA integrity and genomic DNA contamination. The RNA-seq cDNA library of *P. clarkii* ovary was constructed based on the polyA structure of mRNA at the 3′-terminus according to the Hieff NGS MaxUp Dual-mode mRNA Library Prep Kit for Illumina (12301ES96, YEASEN, Shanghai, China) comprising mRNA isolation and preparation, fragmentation, double strand cDNA synthesis, cDNA end repairment and dA-tailing, DNA adapter ligation, and cDNA library amplification by PCR. Purification and fragment size screening of the cDNA library was performed using Hieff NGS DNA Selection Beads (12601ES56, YEASEN, Shanghai, China). The cDNA of the final library was verified by electrophoresis; fragments ranged in size from 300 to 500 base pairs (bp). Finally, the cDNA library was sequenced on an Illumina HiSeq 2500 instrument by Sangon Biotech (Shanghai, China).

### De novo assembly, clustering, and functional annotation

The raw image data files generated by the Illumina HiSeq 2500 instrument were analyzed and converted into raw reads by CASAVA Base Calling. The quality of raw reads was visually evaluated by FastQC software version 0.11.2. The sequence adapters and low quality bases (Quality score < 20) were filtered out, and short length reads (< 35 nt) were removed by Trimmomatic software version 0.36 [50] to obtain clean data. Then, the de novo clean data were assembled into transcripts by Trinity software version 2.4.0 [51], where the parameter min_kmer_cov was set equal to 2, and other parameters were set to default values. The assembled transcripts were de-redundant using RSeQC software version 2.6.1 [52], and the longest transcript in each transcript cluster was taken as a unigene reference sequence for subsequent analysis.

Gene functional annotations were separately performed according to the following databases: NT (NCBI nucleotide sequences, http://ncbi.nlm.nih.gov/), NR (NCBI non-redundant protein sequences, http://ncbi.nlm.nih.gov/), COG/KOG (Clusters of Orthologous Groups of proteins/euKaryotic Ortholog Groups, https://www.ncbi.nlm.nih.gov/COG/) [53], Swiss-Prot (A manually annotated and reviewed protein sequence database), TrEMBL, PFAM (Protein family, http://pfam.xfam.org/) [54], CDD (Conserved Domain Database, https://www.ncbi.nlm.nih.gov/cdd/) [55], GO (Gene Ontology, http://www.geeontology.org), and KEGG (Kyoto Encyclopedia of Genes and Genomes, http://www.kegg.jp) [56]. The annotations of NR, NT, CDD, COG/KOG, Swiss-Prot, TrEMBL, and PFAM were executed by NCBI Blast+ [57]. The GO annotation was harvested based on the results of Swiss-Prot and TrEMBL protein annotation according to the information from Uniprot (http://www.uniprot.org/) [58]. KEGG annotation was performed by KAAS (KEGG Automatic Annotation Server) version 2.1 [59].

### Analysis of differential expression and gene enrichment

Firstly, for sequence evaluation of RNA-seq, Bowtie2 software version 2.3.2 [60] was used to compare effective data of the samples to the transcripts obtained by splicing and to gather statistical mapping information. The duplicate reads and inserted fragment distribution were analyzed by RSeQC software version 2.6.1 [52]. Distribution of gene coverage statistics were performed using BEDTool software version 2.26.0 [61]. Secondly, for analysis of gene expression levels, Salmon software version 0.8.2 [62] and the WGCNA (weighted gene co-expression network analysis) R package version 1.51 [63] were used to calculate the gene expression quantity and to perform gene co-expression analysis, respectively. The comparative analysis of samples and other statistical analyses and exploration in multiple directions were processed based on the expression matrix of the samples. In order to make gene expression levels estimated between different genes and different experiments comparable, we introduced the concept of transcripts per kilobase of exon model per million mapped reads (TPM) to represent the abundance of a transcript and the gene expression level. The formula for TPM was as follows:

$$ {TPM}_i=\frac{X_i}{L_i}\ast \frac{1}{\sum \limits_j\frac{X_j}{L_j}}\ast {10}^6 $$

$$ {X}_i= total\kern0.17em exon\kern0.17em fragment/ reads\kern0.6em {L}_i=\frac{exon\kern0.17em length}{KB} $$

Thirdly, for analysis of differential gene expression, DESeq2 R package version 1.12.4 [64] was utilized to acquire the differentially expressed genes (DEGs) according to the default parameters. The screening conditions were qValue < 0.05 and |Fold Change| > 2 to visualize the results of differential expression model. The DEGs were mapped to the STRING protein-protein interaction network database (http://string-db.org/) [65] for protein interaction network construction. Then, based on the results of differential gene analysis, a Venn diagram and heat map were drawn, and a cluster analysis was carried out. Finally, for gene enrichment analysis, topGO R package version 2.24.0 [66] was used for analysis of GO enrichment, and the clusterProfiler R package version 3.0.5 [67] was used for KEGG pathway and KOG category enrichment analysis, then draw the associated analysis network diagram.

### Real-time quantitative PCR (RT-qPCR) for DEGs validation

In order to validate the expression profile of DEGs from RNA-seq, we chose nine DEGs for further RT-qPCR detection. One microgram of total RNA from each sample was reverse transcribed into cDNA by Maxima Reverse Transcriptase (EP0743, ThermoFisher Scientific, USA) according to the manufacturer’s instructions. The RT-qPCR was conducted in a final volume of 20 μL that consisted of 10 μL of SYBR Green PCR Master Mix (#4309155, ThermoFisher Scientific, USA), 0.4 μL of forward primer (10 μM), 0.4 μL of reverse primer (10 μM), 2 μL of cDNA template and 7.2 μL of ddH_2_O. The RT-qPCR was performed in an ABI StepOne plus instrument (ABI, California, USA). The reaction conditions were as follows: denaturation at 95 °C for 10 min; followed by 45 cycles of 95 °C for 15 s, 60 °C for 30 s; the melt curve was read according to instrument guidelines. Glyceraldehyde-3-phosphate dehydrogenase (GAPDH) was used as an internal reference gene for normalization. All primers used for RT-qPCR are shown in Supplementary Table S1. The fold changes of target genes between each comparison group were calculated according to the relative quantitative 2^-△△Ct^ method formula. The RT-qPCR experiment was performed triplicate, and the data were shown as mean ± S.D. The statistical analysis was carried out based on one-way ANOVA.

## Results

### Identification of different ovarian developmental stages

**Fig. 1:**
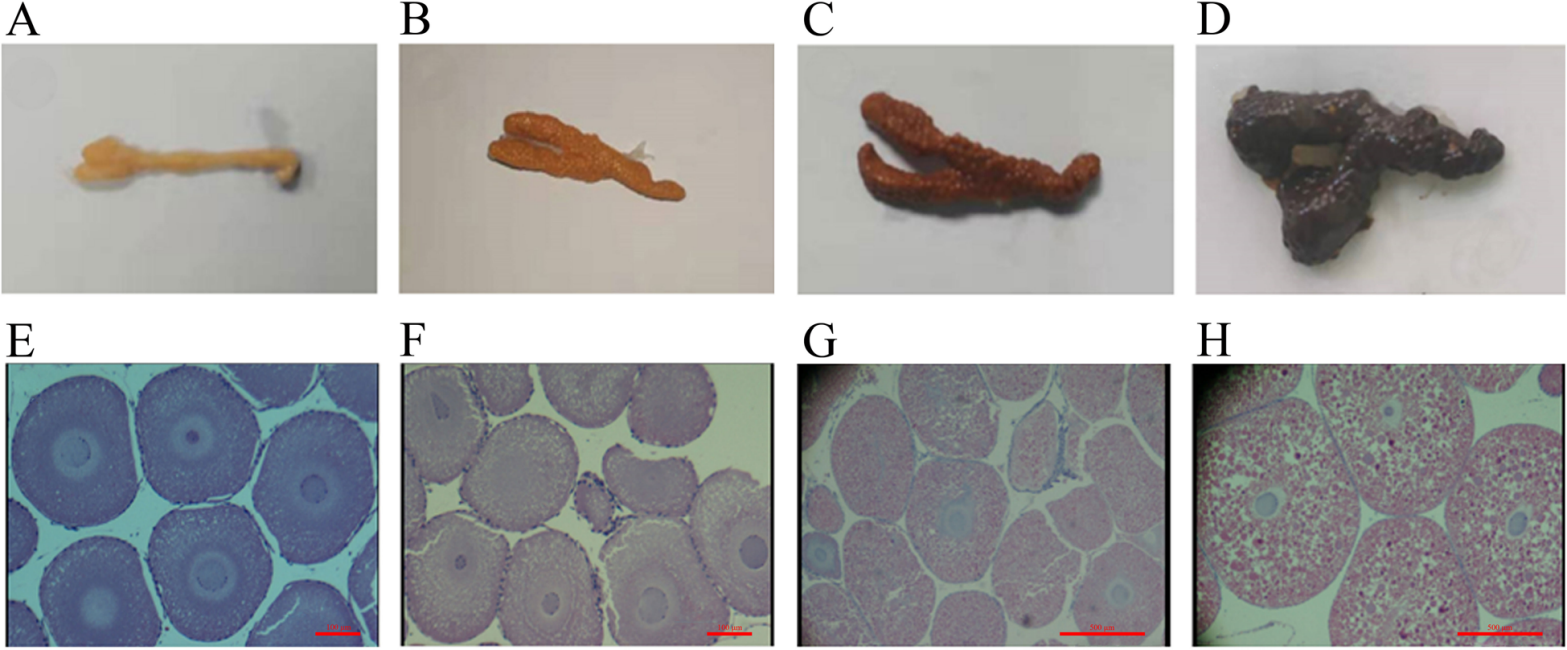
Identification of ovaries at different developmental stages of *P. clarkii*. **a**-**d**: The morphology and color of ovaries at different stages by photograph; **a**: The morphology and color of the ovary at stage I, **b**: The morphology and color of the ovary at stage II, **c**: The morphology and color of the ovary at stage III, **d**: The morphology and color of the ovary at stage IV. E-H: The histomorphology of ovaries at different stages by HE staining; **e**: The oocytes at stage I, bar = 100 μm, **f**: The oocytes at stage II, bar = 100 μm, **g**: The oocytes at stage III, bar = 500 μm, **h**: The oocytes at stage IV, bar = 500 μm

The female crayfish were captured monthly from May to September, and the ovaries were collected and photographed to record the morphology and color (Fig. 1a-d). According to the color and size, we divided the ovaries into four stages, the previtellogenic stage (stage I), the early vitellogenic stage (stage II), the middle vitellogenic stage (stage III), and the mature stage (stage IV). The stage I ovary was yellowish white and thin, the ovary outer membrane was thick, and the egg particles were inconspicuous (Fig. 1a); the stage II ovary was yellow and became larger, and the outer membrane became thinner, the egg particles were obvious and were the size of rice grains (Fig. 1b); the stage III ovary was light brown and larger than the stage II ovary, the size of egg particles continuously increased, and the particles were closely arranged (Fig. 1c); the stage IV ovary was dark brown or black, and the volume was extremely inflated, the eggs were plump and discrete (Fig. 1d). In addition, we detected the histomorphology of the ovary by HE staining to confirm the four stages (Fig. 1e-h). The oocytes at stage I were small and roundish, the diameter was 100–200 μm, and the nucleus and cytoplasm were blue by HE staining (Fig. 1e); most of the oocytes at stage II were oval or subrotund and 200–500 μm in diameter, the cytoplasm was stained in most red and partially blue (Fig. 1f), indicating that yolk granules began appearing; most of the oocytes at stage III were more than 500 μm in diameter, the cytoplasm was obviously red by HE staining, and the number of yolk granules increased and the size became larger (Fig. 1g); The oocytes at stage IV were the largest (> 1000 μm) and were full of yolk granules that were larger than those at other stages and were dark red (Fig. 1h). These results revealed that the sizes of the oocytes and yolk granules increased as the ovary developed.

### Assembly and information analysis of transcriptome data

There were 12 ovary samples of *P. clarkii* in four different developmental stages that were subjected to RNA-seq, including stage I (I_7, I_19 and I_20, named group A), stage II (II_17, II_27 and II_30, named group B), stage III (III_33, III_49 and III_52, named group C), and stage IV (IV_35, IV_36 and IV_37, named group D). Analyses were done in triplicate for each stage. The total raw read counts of all 12 samples ranged from 43,433,438 to 64,090,726 (Supplementary Table S2). The GC base ratios of raw data were between 45.31 and 51.71%, with an average of was 47.2%. Except for the sample IV_36 (51.71%), the GC contents of the remaining 11 samples were less than 50% (Supplementary Table S2 and Supplementary Figure S1), indicating that the GC ratio of the transcripts was less than the AT ratio in the ovary of *P. clarkii*. After quality control by removing adapters and low-quality bases (Quality score < 20), the total clean read counts of all 12 samples ranged from 42,013,648 to 62,220,956, while the total clean base counts ranged from 6,118,532,265 bp to 9,054,802,655 bp, and the Q30 base ratios (the proportion of nucleotides with quality value ≥30) were 94.52–95.58%. The GC base ratios were 45.08–51.35%, and the average was 46.99% (Supplementary Table S3), consistent with the raw read data.

**Fig. 2:**
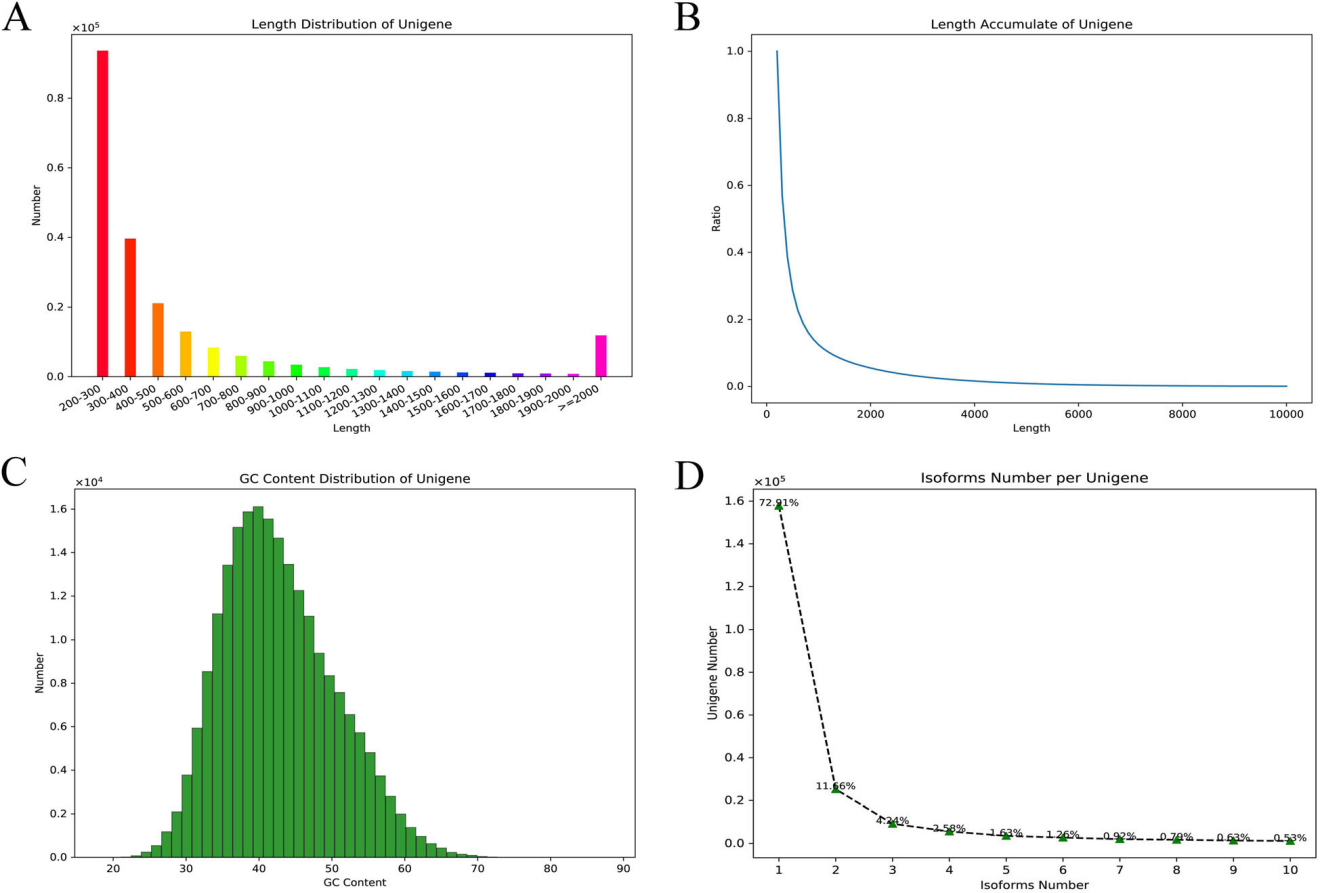
The assembly and information of the transcriptome data of the *P. clarkii* ovary. **a**: The length distribution of unigenes after assembly, the abscissa represents the length range of unigenes, the ordinate represents the number of unigenes corresponding to the length. **b**: The length accumulate of unigenes after assembly, the abscissa represents the length of unigene, the ordinate represents the ratio of unigenes which were more than the corresponding length. **c**: The GC content distribution and the corresponding numbers of unigenes. **d**: The distribution of isoforms number per unigene

There were 445,326 transcripts and 216,444 unigenes obtained after assembly, and the N50 lengths were 1858 bp and 912 bp, respectively (Table 1). All of the transcripts and unigenes ranged from 201 bp to 20,027 bp, and there were 62,141 and 26,934 unigenes that were ≥ 500 bp and ≥ 1000 bp, respectively (Table 1). The length distribution of the sequences showed that most of the transcripts and unigenes were less than 1000 bp (Supplementary Figure S2A and Fig. 2a and b), accounting for 77.94 and 87.56%, respectively. The GC content distribution demonstrated that the GC ratios of most transcripts and unigenes were less than 50% (Supplementary Figure S2C and Fig. 2c) being mainly distributed around 40%, coinciding with the raw and clean read data (Supplementary Table S2 and Supplementary Table S3). The isoform (also referred to as the transcript) number of each unigene indicated that 72.91% of the unigenes had only one isoform, and 11.66, 4.24, 2.58, 1.63, and 1.26% of the unigenes had 2, 3, 4, 5, and 6 isoforms, respectively (Fig. 2d).

**Table 1:** The assembly result of transcript and unigene

	No.	≥500 bp	≥1000 bp	N50 (bp)	N90 (bp)	Max Length (bp)	Min Length (bp)	Total Length (bp)	Average Length (bp)
Transcript	445,326	168,661	98,221	1858	292	20,027	201	381,942,933	857.67
Unigene	216,444	62,141	26,934	912	253	20,027	201	133,770,495	618.04

N50/N90: The length at 50%/90% of total length of the assembly transcript, which was the length of the cumulative transcript in the order from large length to small length

### Overall functional annotation and analysis

**Fig. 3:**
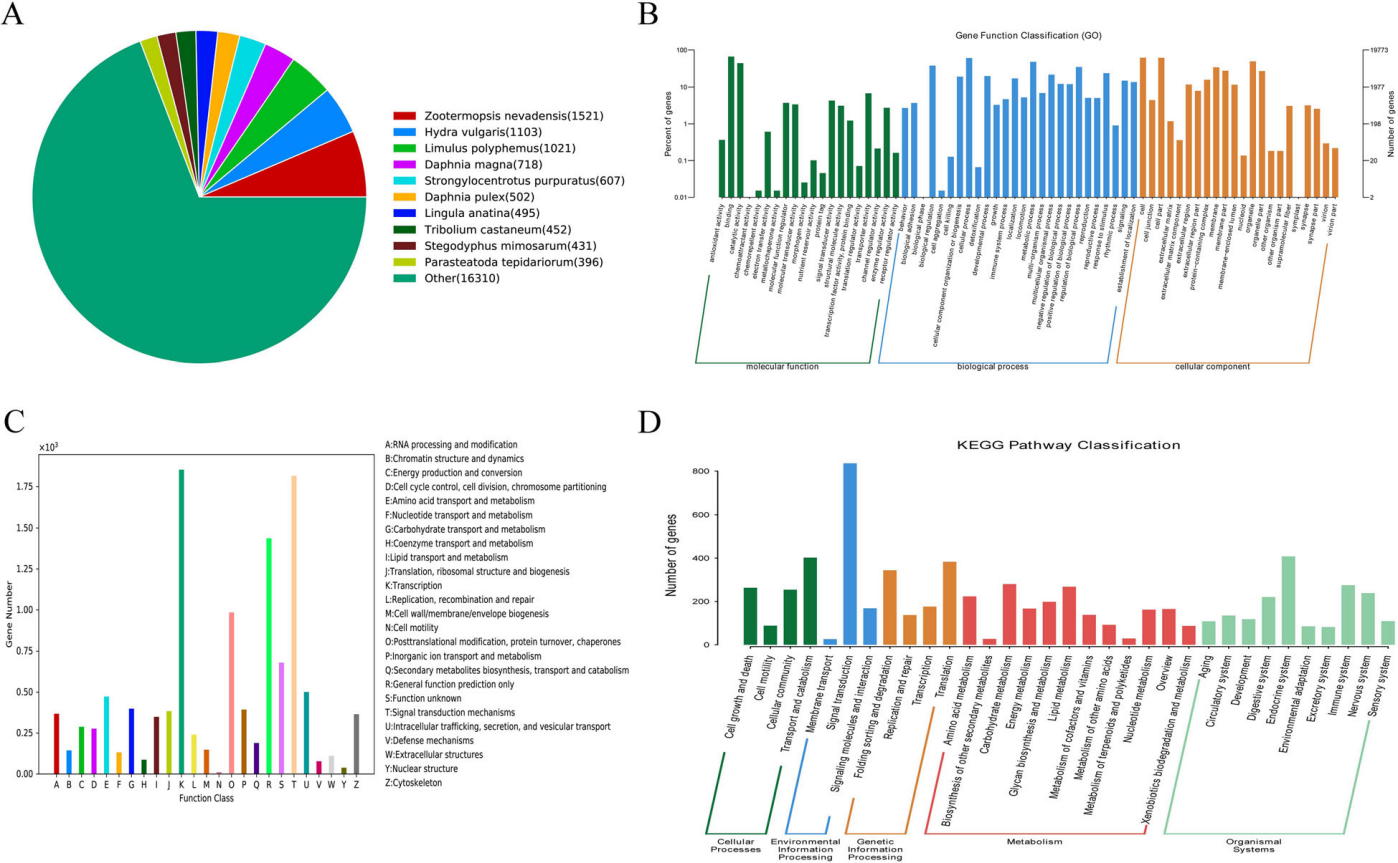
The overall functional annotation of the assembled unigenes. **a**: The distribution of matched species of the unigenes according to the NR database. **b**: The overall GO classification annotation of the unigenes. **c**: The overall KOG functional classification of the unigenes. **d**: The overall KEGG pathway classification of the unigenes

All 216,444 unigenes of the *P. clarkii* ovary were searched in the nine public databases NT, NR, KOG, Swiss-Prot, TrEMBL, PFAM, CDD, GO, and KEGG using a cut-off E-value of 10^− 5^. There were 8872 (4.1%), 23,683 (10.94%), 10,520 (4.86%), 14,913 (6.89%), 25,706 (11.88%), 8504 (3.93%), 12,005 (4.86%), 19,773 (9.14%), and 3560 (1.64%) annotations, respectively (Table 2). Of these, 32,599 (15.06%) and 1065 (0.49%) unigenes were annotated in at least one database and annotated in all databases, respectively (Table 2), indicating that most of the unigenes were not annotated. By comparison with the NR database, the transcript similarity between *P. clarkii* and similar species and the functional information of the homologous transcripts could be obtained (Supplementary Table S4). In the NR blast result, 1521 unigenes from the RNA-seq data in this study were best matched with the genes of *Zootermopsis nevadensis*, followed by *Hydra vulgaris* (1103 unigenes) and *Limulus polyphemus* (1021 unigenes) (Supplementary Table S5 and Fig. 3a). As a species in the phylum Arthropoda, the sequences of *P. clarkii* were similar to known sequences of other species of Arthropoda and Hydrozoa. Among the blast species, only 256 unigenes from RNA-seq data were matched with known genes of *P. clarkii* from the NR database (Supplementary Table S5). When the duplicate genes were removed, only 113 genes were matched (Supplementary Table S4), indicating that most of the genes expressed in the ovary of *P. clarkii* remain largely unknown.

**Table 2:** The summary of gene annotation

Database	Number of genes	Percentage (%)
Annotated in NT	8872	4.1
Annotated in NR	23,683	10.94
Annotated in KOG	10,520	4.86
Annotated in Swissprot	14,913	6.89
Annotated in TrEMBL	25,706	11.88
Annotated in PFAM	8504	3.93
Annotated in CDD	12,005	5.55
Annotated in GO	19,773	9.14
Annotated in KEGG	3560	1.64
Annotated in at least one database	32,599	15.06
Annotated in all database	1065	0.49
Total genes	216,444	100

In the GO functional classification analysis, a total of 19,773 unigenes were annotated to at least one GO term; all the GO annotated unigenes were classified into 68 GO terms belonging to the three main categories of molecular function, biological process, and cellular components (Fig. 3b and Supplementary Table S6). Within the molecular function category, the binding (13,063 unigenes, 66.04%) and catalytic activity (8656 unigenes, 43.78%) were the most dominant subcategories. The biological process category was the most frequently annotated, and the cellular process (11,953 unigenes, 60.45%), metabolic process (9391 uningenes, 47.49%), biological regulation (7393 unigenes, 37.39%), and regulation of biological process (6816 unigenes, 34.47%) were the most common subcategories. The cellular component was the second most frequently annotated category, and the cell (12,162 unigenes, 61.51%), cell part (12,144 unigenes, 61.42%), organelle (9691 unigenes, 49.01%), and membrane (6680 unigenes, 33.78%) were the most common subcategories (Fig. 3b). In the biological process category, the subcategory of developmental process had 3930 (19.88%) unigenes associated with ovarian development, and the subcategories of reproduction and reproductive process had 1002 (5.07%) and 997 (5.04%) unigenes, respectively, associated with reproduction (Fig. 3b). In order to understand the functions of lineal homologous genes of *P. clarkii* from other species, the KOG analysis was performed and the 10,520 annotated unigenes were divided into 25 functional categories. The results showed that the cluster of transcription (1854 unigenes, 17.62%) was the most dominant in the ovary of *P. clarkii*, followed by signal transduction mechanisms (1817 unigenes, 17.27%), general function prediction only (1437 unigenes, 13.66%), and posttranslational modification, protein turnover, chaperones (984 unigenes, 9.35%) (Fig. 3c and Supplementary Table S7). In addition, to understand the biological pathways of the unigenes, 3560 unigenes annotated in the KEGG database were analyzed and were assigned to 275 pathways, the number of unigenes in each pathway ranged from 1 to 135; the PI3-Akt signaling pathway (ko04151), endocytosis (ko04144), purine metabolism (ko00230) and RNA transport (ko03013) were the distinct pathways (Supplementary Table S8). In the KEGG pathway, the cellular processes, environmental information processing, genetic information processing, metabolism and organismal systems were identified, and these comprised 4, 3, 4, 12, and 10 subgroups, respectively, of which signal transduction, endocrine system, transport and catabolism, and translation were prominent (Fig. 3d and Supplementary Table S8).

### Correlation analysis of *P. clarkii* ovarian samples at different stages

**Fig. 4:**
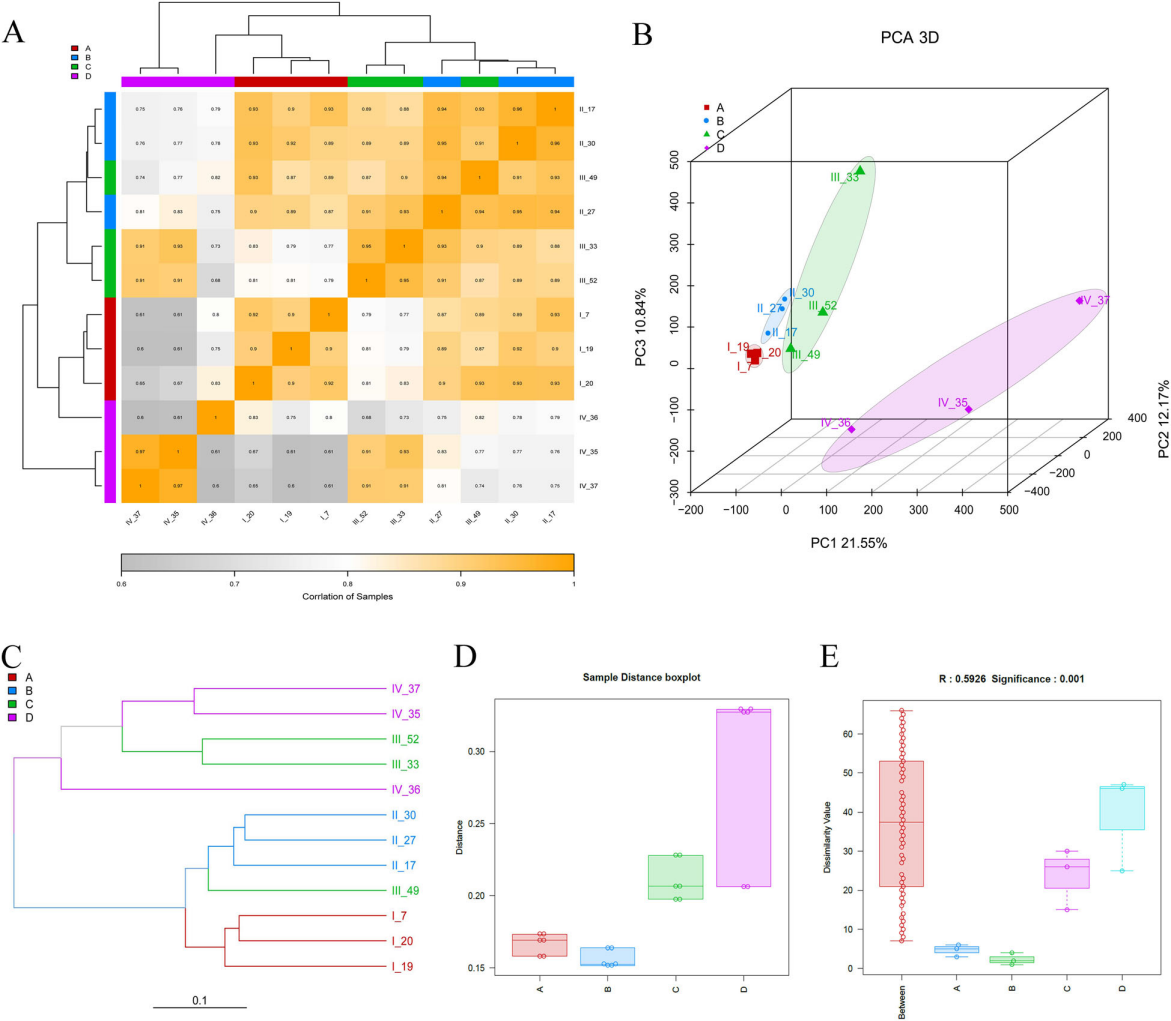
The correlation of ovary samples at different stages of *P. clarkii* based on the RNA-seq analysis. **a**: The heatmap of the Pearson coefficients among all the ovary samples. The more grey the color of the block, the lower correlation between two samples; the more yellow the color, the higher correlation between two samples. **b**: The Principal Component Analysis (PCA) of all the twelve ovary samples. **c**: Hierarchical clustering based on the distances among the twelve samples. The length of the branch represents the distance between the two samples, the more similar the samples, the closer they get. **d**: The sample distance boxplot of the four stages. **e**: The analysis of similarity (Anosim) boxplot among different groups. Note: The alphabet of **a**, **b**, **c** and **d** in the graph represent the stage I, stage II, stage III and stage IV, respectively

The 12 ovaries of *P. clarkii* were divided into four stages according to color, size, and histomorphology (Fig. 1). The stages were defined as stage I (I_7, I_19 and I_20), stage II (II_17, II_27 and II_30), stage III (III_33, III_49 and III_52), and stage IV (IV_35, IV_36 and IV_37). After the RNA-seq analysis, the repeatable correlation among all the samples was performed, and the Pearson, Kendall, and Spearman coefficient were calculated. The Pearson coefficients between different samples within one group of stages I-III were greater than 0.8, representing extremely strong positive correlations; the Pearson coefficients between each sample of stage IV were greater than 0.6, showing strong positive correlations (Supplementary Figure S3). The Pearson coefficients demonstrated that stage IV was the most distant from stage I and stage II, and stage II was similar to stage III (Fig. 4a and Supplementary Figure S4A). IV_36 was different from IV_35 and IV_37 at stage IV (Fig. 4a and Supplementary Figure S4B). The Principal Component Analysis (PCA) showed that the samples from one stage could be distinguished from other stages (Fig. 4b and Supplementary Figure S4C). The algorithm of Bray Curtis was used to calculate the distance between different samples, and the Bray tree was constructed according to the distances. The results showed that stage I and stage II were in the same cluster, and stage III (III_33 and III_52) and stage IV were in another cluster, while III_49 (stage III) was in the stage II cluster (Fig. 4c). The Bray tree of the merged group also showed that stage IV was the most distant from other stages, and stage II and stage III were in the same cluster (Supplementary Figure S4D). In order to compare the distribution difference of distances within and among different groups, distance boxplots were drawn; these showed that the differences in distance between stages I, II and III were small, but there was one sample (IV_36) that was far from the other two samples in stage IV (Fig. 4d). To determine whether the grouping was meaningful, a non-parametric analysis of similarity (Anosim) was performed. If the R value is near 1, the difference among groups is greater than that within groups; in contrast, when the R value is near 0, there is no significant difference within and among groups. The R value from the Anosim among the four groups was 0.5926 (*p* < 0.001) (Fig. 4e), indicating that the difference among groups was greater than that within groups. These results demonstrated that the grouping of four stages in this study was reliable, and the characteristics of the four stages of *P. clarkii* ovaries were different from each other.

### Differential expression analysis of *P. clarkii* ovarian samples at different stages

The 216,444 unigenes were obtained from the twelve ovarian samples of *P. clarkii*, and the TPM values and annotations of the highly expressed unigenes are listed in Supplementary Table S9. The TPM values represent the expression levels of genes. The four stages were employed for pairwise comparisons using five comparison groups, including stage II_vs_stage I (B_vs_A), stage III_vs_stage I (C_vs_A), stage IV_vs_stage I (D_vs_A), stage III_vs_stage II (C_vs_B), and stage IV_vs_stage III (D_vs_C). The TPM density distribution revealed that log_2_(TPM) values of the twelve ovarian samples mainly ranged from − 5 to 5, with only a small fraction in the range − 15 to − 12. The log_2_(TPM) values of IV_35, IV_36, and IV_37 of group D were the largest, indicating that the gene expression of stage IV was different from those of other stages (Supplementary Figure S5A). The TPM density distribution between groups demonstrated that the gene expression of one group was different from other groups, and the order of expression level was D > C > B > A (Supplementary Figure S5B-F).

**Fig. 5:**
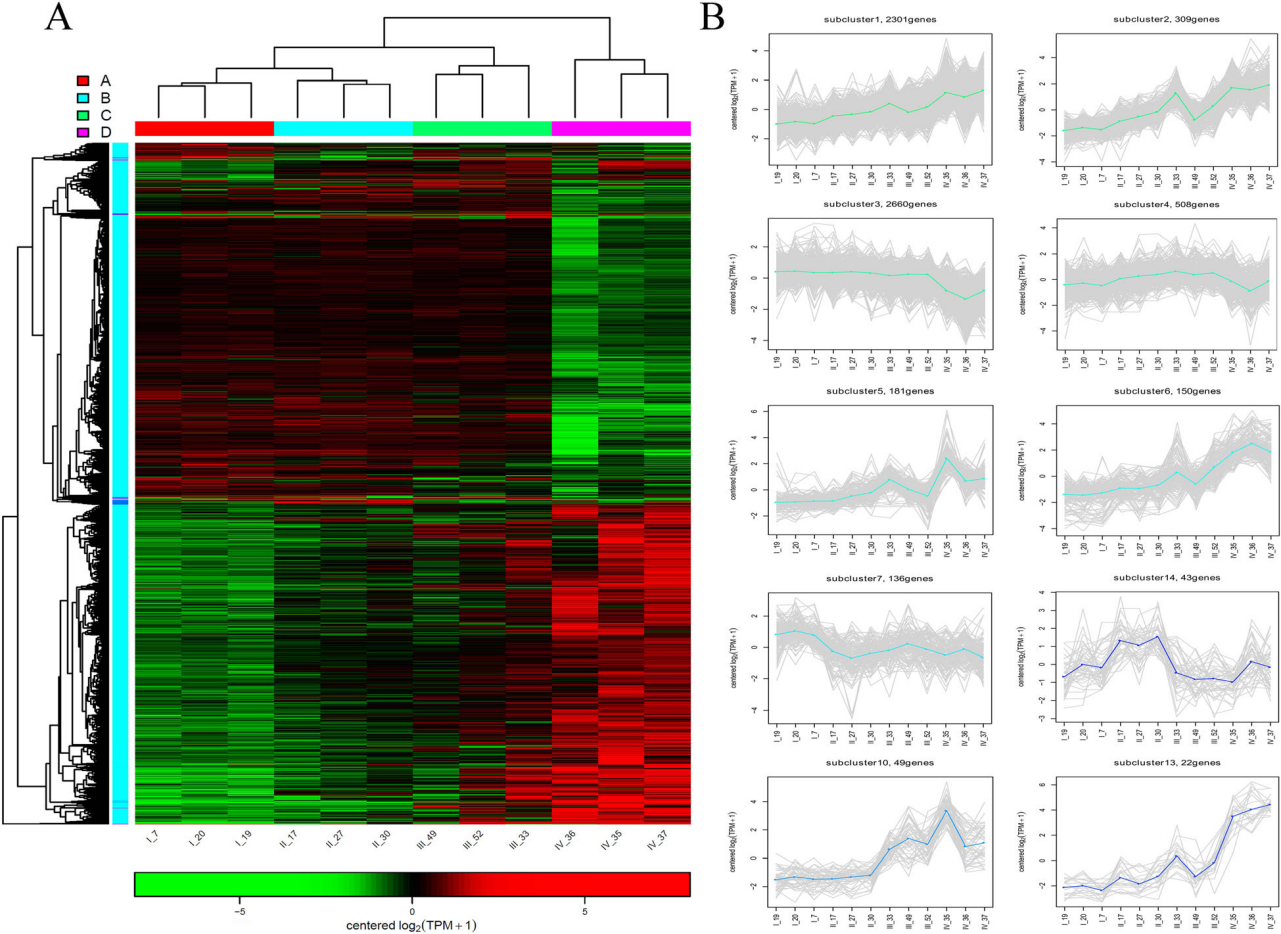
The gene expression pattern of DEGs of twelve ovary samples of *P. clarkii*. **a**: The cluster heatmap of DEGs among the twelve ovarian samples. Groups A, B, C and D represent stage I, stage II, stage III and stage IV, respectively. The green color in the matrix represents downregulation of unigene, the red color in the matrix represents upregulation of unigene. **b**: The gene expression patterns within regular subclusters

**Fig. 6:**
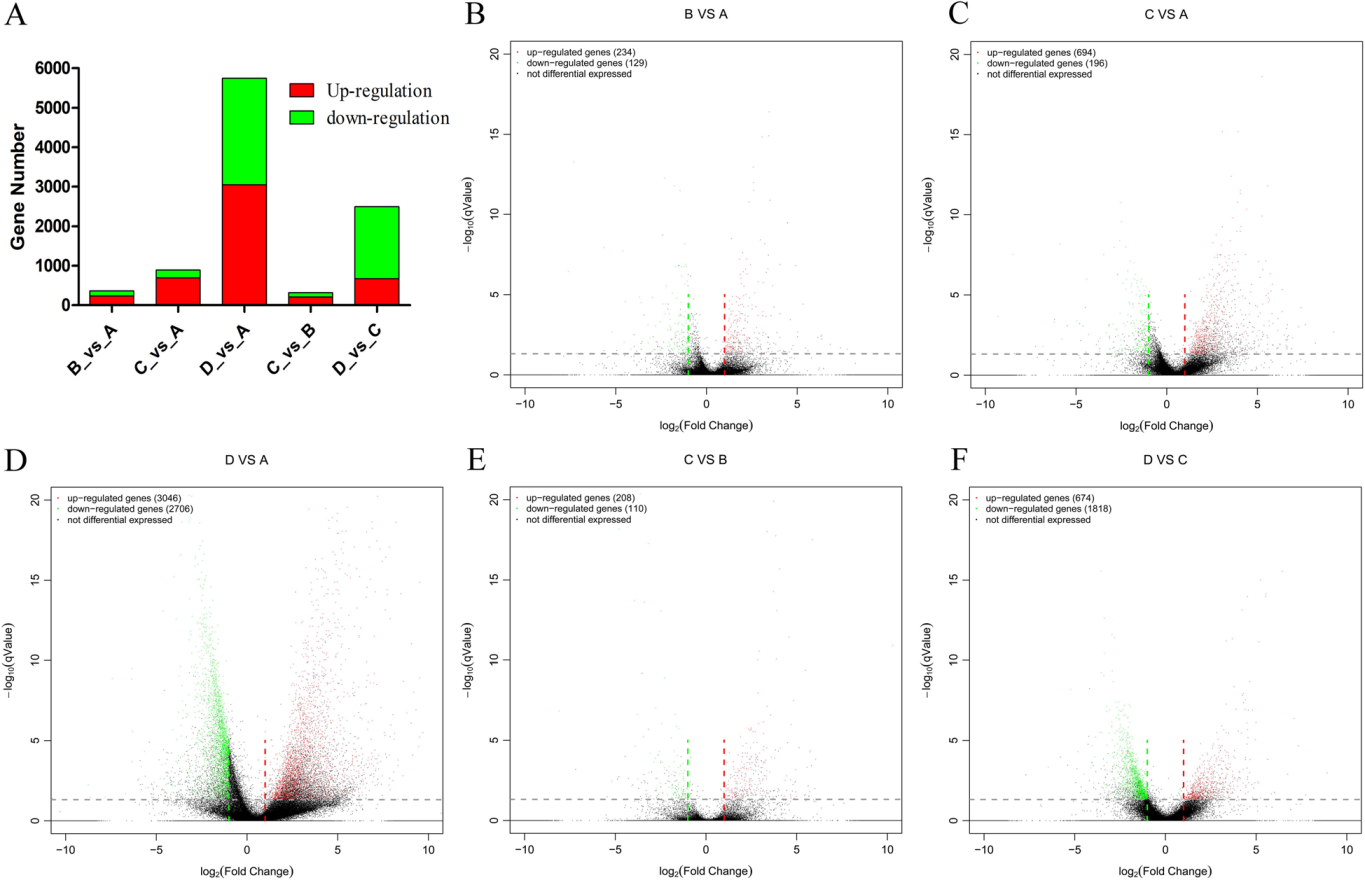
The visualization analysis of expression differences among different comparisons. **a**: The barplot of the DEGs in five comparisons. B_vs_A is stage II_vs_stage I, C_vs_A is stage III_vs_stage I, D_vs_A is stage IV_vs_stage I, C_vs_B is stage III_vs_stage II, D_vs_C is stage IV_vs_stage III. **b**: The DEGs volcano of stage II_vs_stage I. **c**: The DEGs volcano of stage III_vs_stage I. **d**: The DEGs volcano of stage IV_vs_stage I. **e**: The DEGs volcano of stage III_vs_stage II. **f**: The DEGs volcano of stage IV_vs_stage III. One dot in the volcano represents one gene, red dots represent up-regulated genes and green dots represent down-regulated genes, black dots indicate the undifferentiated genes. The smaller the qValue, the larger the -log (qValue), the more significant the difference of the DEGs

**Fig. 7:**
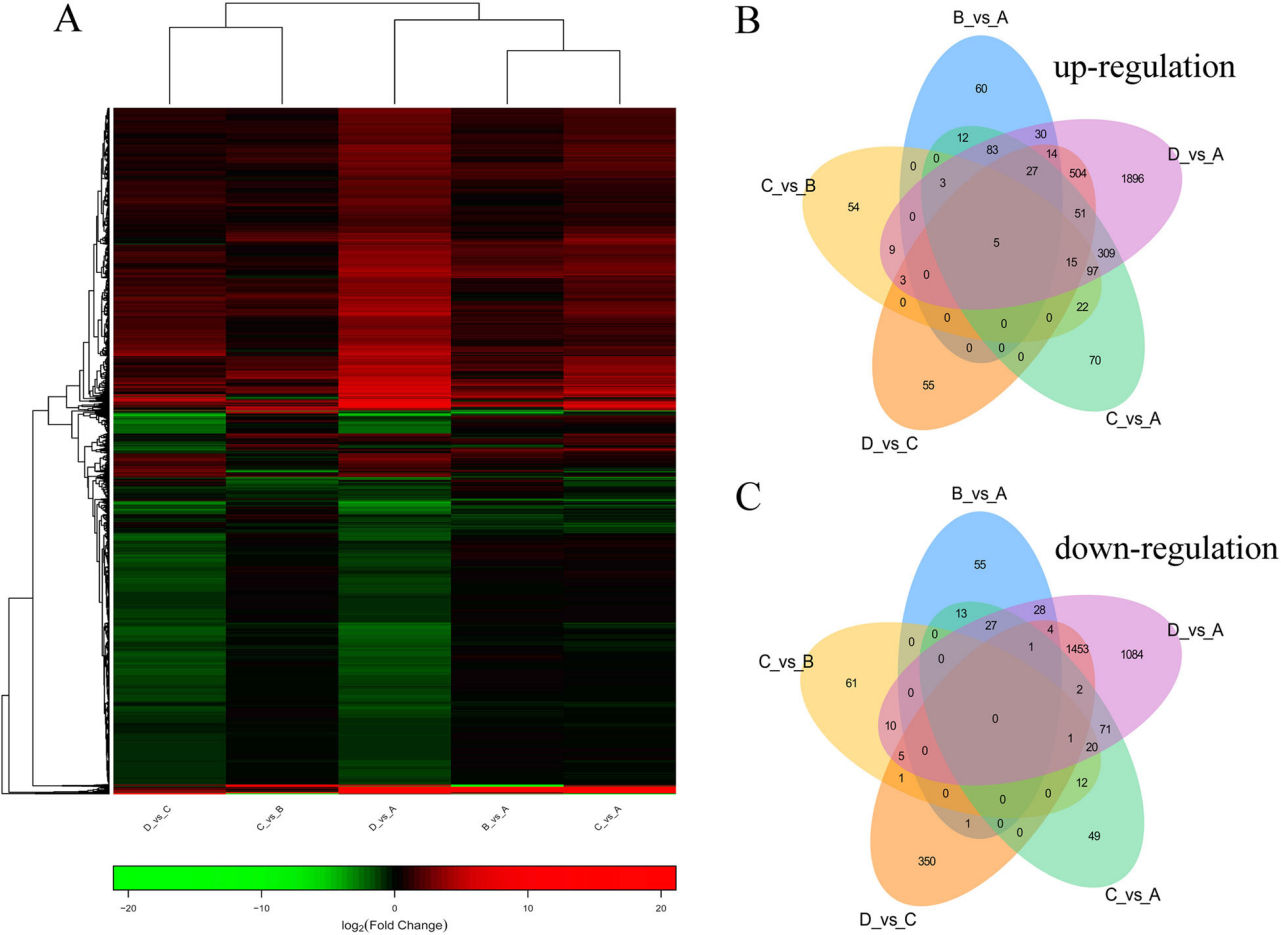
The analysis of co-expressive DEGs among different comparison groups. **a**: The heatmap of the DEGs among five comparisons. The green color in the matrix represents down-regulated genes, the red color in the matrix represents up-regulated genes. **b**: The venn diagram of the up-regulated DEGs among the five comparisons. **c**: The Venn diagram of the down-regulated DEGs among the five comparisons. Different comparison groups are represented in different colors, the overlapping region represents the number of DEGs shared by different comparison groups, the nonoverlapping region represents the number of DEGs belonging to only one comparison group

Further, the cluster heatmap of DEGs based on the twelve ovarian samples was drawn (Fig. 5a). The results illustrated that the expression characteristics of IV_35, IV_36, and IV_37 in group D were clearly distinguished from the other samples. The expression characteristics of II_17, II_27, and II_30 in group B were similar with III_33, III_49, and III_52 in group C. According to the expression pattern in the cluster heatmap, all the DEGs were categorized into 30 subclusters (Fig. 5a), including 10 subclusters with regular trends (Fig. 5b) and 20 subclusters with irregular trends (Supplementary Figure S6). Among the regular subclusters, the expression trend of subcluster 1 (2301 genes) continuously increased from stage I to stage IV; the subcluster 3 (2660 genes) showed that the gene expression from stage I to stage III presented as a horizontal line, while being sharply decreased at stage IV; the genes in subcluster 7 (136 genes), subcluster 14 (43 genes), subcluster 10 (49 genes), and subcluster 13 (22 genes) were uniquely highly expressed in stage I, stage II, stages III-IV, and stage IV, respectively (Fig. 5b), illustrating that these genes had stage specificity. In addition, the number of DEGs between different groups revealed that the number of DEGs in D_vs_A was the highest, followed by D_vs_C and C_vs_A. The numbers of DEGs in C_vs_B and B_vs_A were relatively low (Fig. 6a and Supplementary Table S10). The volcano plot listed the number of DEGs in five comparison groups, the comparisons of B_vs_A, C_vs_A, D_vs_A, C_vs_B, and D_vs_C had 234, 694, 3046, 208, and 674 significantly up-regulated genes, respectively, and 129, 196, 2706, 110, and 1818 significantly down-regulated genes, respectively (Fig. 6b-f). Simultaneously, the DEGs heatmap of five comparison groups also confirmed that the difference between group D and group A was the most conspicuous, followed by between group D and group C (Fig. 7a). According to the information of DEGs, the Venn diagram was conducted, which could observe the DEGs distribution among different comparison groups, in the up-regulated DEGs, there were five core genes up-regulated in all five comparison groups (Fig. 7b), but no core gene was down-regulated in any of the five comparison groups (Fig. 7c). These data demonstrated that the expression patterns among stages I, II, III, and IV of *P. clarkii* ovaries were different, and the number of DEGs continuously increased with the development of the ovary.

### Functional annotation of differentially expressed genes

**Fig. 8:**
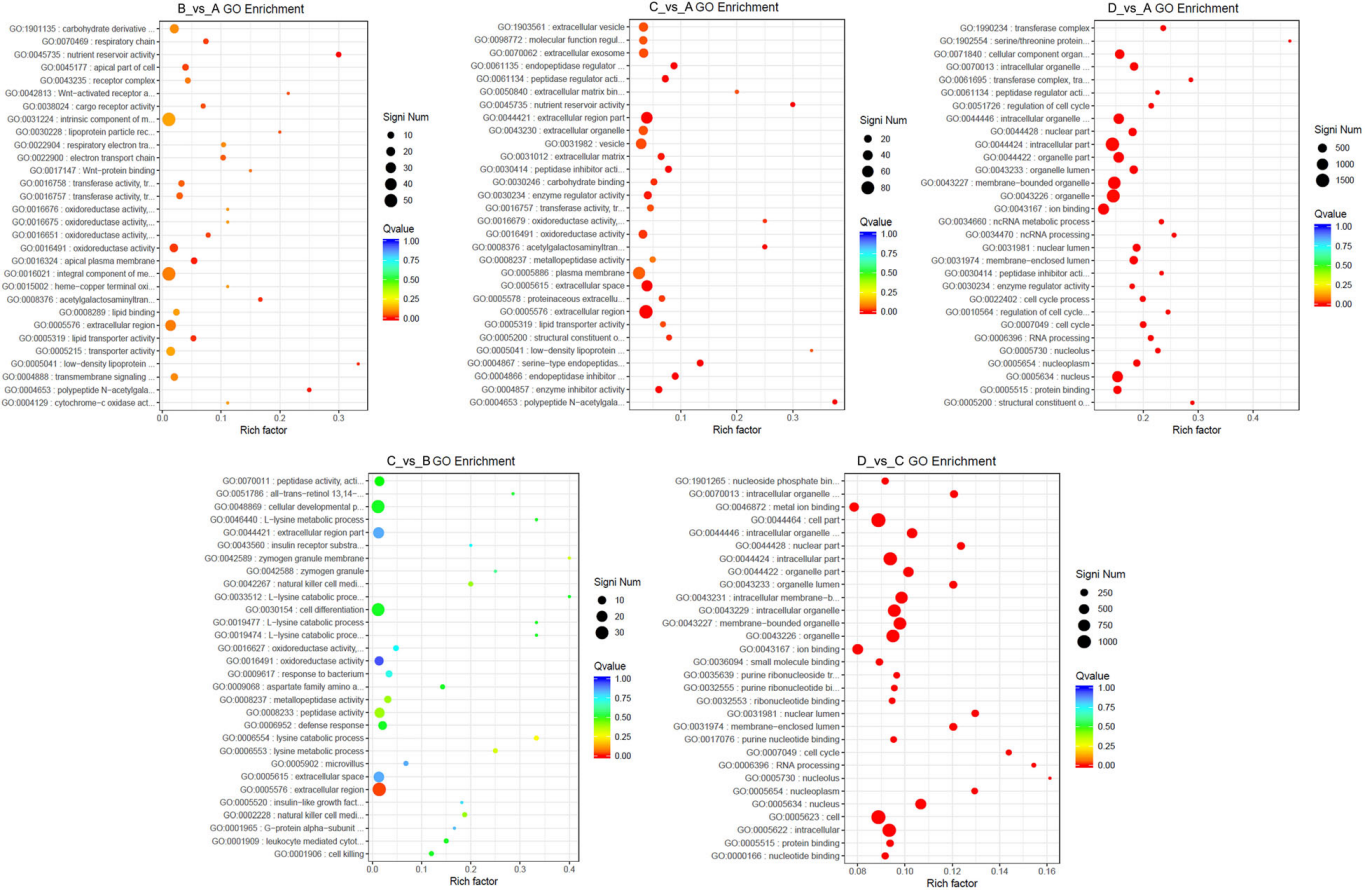
The analysis of GO functional enrichment. The ordinate represents the top thirty enriched GO terms, the Rich factor is the proportion of the DEGs annotated in the GO term to the total annotated genes in the GO term. The more red the color of the Q value, the more significant the enrichment of the GO term. The significant number of DEGs in the GO term is represented by the size of the circle

After selecting the DEGs, the distributions and enrichments of DEGs in the GO and KEGG functional categories were performed to discover the gene sets with differential expression levels. The enrichment analysis could identify the biological pathways that were most relevant to the biological phenomena, while the subcategory is thought to be significant enrichment if the Q value is less than 0.05. The GO classification of DEGs showed the distribution among three main categories (biological process, cellular component, and molecular function), the results indicated that the numbers of DEGs in most of the subcategories for the B_vs_A and C_vs_B comparison groups were relatively low; the numbers of DEGs in the C_vs_A and D_vs_C comparison groups were considerably increased, and the DEGs number in D_vs_A comparison group was obviously greater than in other groups (Supplementary Figure S7). The DEGs of biological process in all comparison groups were mainly distributed in the terms of cellular process, metabolic process, and biological regulation; the DEGs of cellular component in all comparison groups mainly reflected on the terms of cell, organelle and membrane; the DEGs of molecular function in all comparison groups mainly located in the terms of binding, catalytic activity, and transporter activity (Supplementary Figure S7 and Supplementary Table S11). Furthermore, the DEGs of most of the terms in B_vs_A, C_vs_A and C_vs_B comparisons were dominated by up-regulation, while the DEGs in D_vs_A and D_vs_C comparisons were dominated by down-regulation (Supplementary Table S11), illustrating that the some GO terms increased from stage I to stage III, but decreased at stage IV. Interestingly, the nutrient reservoir activity of molecular function in B_vs_A and C_vs_A comparisons had six up-regulated DEGs and was significant (Q value < 0.05) in the functional enrichment, but had no DEG in other comparisons (Supplementary Table S11), implying that the nutrient reservoir activity at stage II played a foreshadowing role for subsequent ovarian development. Within the biological process category, 1002 unigenes associated with reproduction (GO:0000003) were annotated, and the B_vs_A, C_vs_A, D_vs_A, C_vs_B, and D_vs_C comparisons had 13, 30, 153, 12, and 81 significant unigenes, respectively, but the Q values in all comparisons were greater than 0.05 (Supplementary Table S11 and S12). The GO enrichment analysis demonstrated that the B_vs_A comparison had some significantly enriched GO terms, but the number was less than that of the C_vs_A comparison, while the C_vs_B comparison had only one significantly enriched GO term (extracellular region, GO:0005576). The GO enrichment of D_vs_A was similar to D_vs_C (Fig. 8 and Supplementary Table S12). The B_vs_A, C_vs_A, D_vs_A, C_vs_B, and D_vs_C comparisons possessed 15, 29, 128, 1, and 133 significant GO terms, respectively (Supplementary Table S12). These data demonstrated that the difference between stage IV and stage III was the most obvious, and cell (GO:0005623), intracellular (GO:0005622), and organelle (GO:0043226) were the prominent GO terms, implying that the unigenes in these GO terms participated in the ovary maturation at stage IV.

**Fig. 9:**
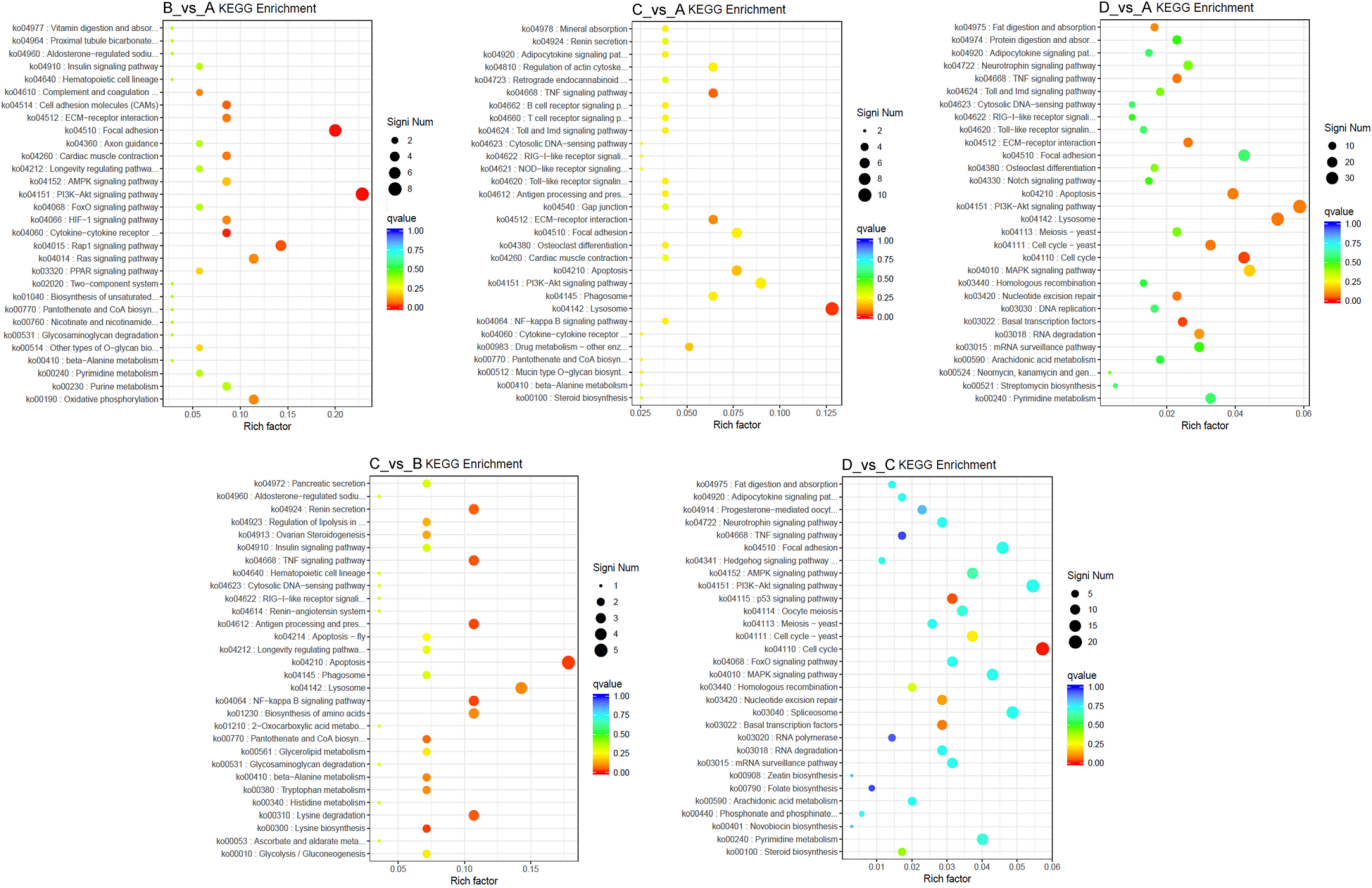
The analysis of KEGG pathway functional enrichment. The ordinate represents the top thirty enriched KEGG pathways, the Rich factor is the proportion of the DEGs annotated in the KEGG pathway to the total annotated genes in the KEGG pathway. The more red the color of the Q value, the more significant the enrichment of the KEGG pathway. The significant number of DEGs in the KEGG pathway is represented by the size of the circle

In addition, the KEGG classification of DEGs displayed the distribution of five main categories (cellular processes, environmental information processing, genetic information processing, metabolism, and organismal systems) (Supplementary Figure S8 and Supplementary Table S13). The subgroup of signal transduction belonging to environmental information processing was relatively prominent in all five comparisons; the significant numbers of each subgroup in D_vs_A and D_vs_C were greater than in the other three comparison groups, as in the analysis for the corresponding GO categories. However, the KEGG enrichment analysis disclosed that seven significantly enriched subgroups (apoptosis, ko04210; lysine biosynthesis, ko00300; NF-kappa B signaling pathway, ko04064; antigen processing and presentation, ko04612; TNF signaling pathway, ko04668; lysine degradation, ko00310; and renin secretion, ko04924) were observed in C_vs_B comparison group, more than in the other four comparison groups, indicating that these subgroups were responsible for the transition from stage II to stage III. The B_vs_A, C_vs_A, D_vs_A, and D_vs_C comparison groups had only two (PI3K-Akt signaling pathway, ko04151; focal adhesion, ko04510), one (lysosome, ko04142), two (basal transcription factors, ko03022; cell cycle, ko04110), and two (cell cycle, ko04110; p53 signaling pathway, ko04115) significantly enriched subgroups, respectively (Fig. 9 and Supplementary Table S14). These results revealed that the early development of *P. clarkii* ovary was mainly associated with the PI3K-Akt signaling pathway and focal adhesion, the middle development of the ovary was related to apoptosis, lysine biosynthesis, and NF-kappa B signaling pathway, and the late development of the ovary was involved with cell cycle and p53 signaling pathway.

### RT-qPCR confirmation of RNA-seq data

**Fig. 10:**
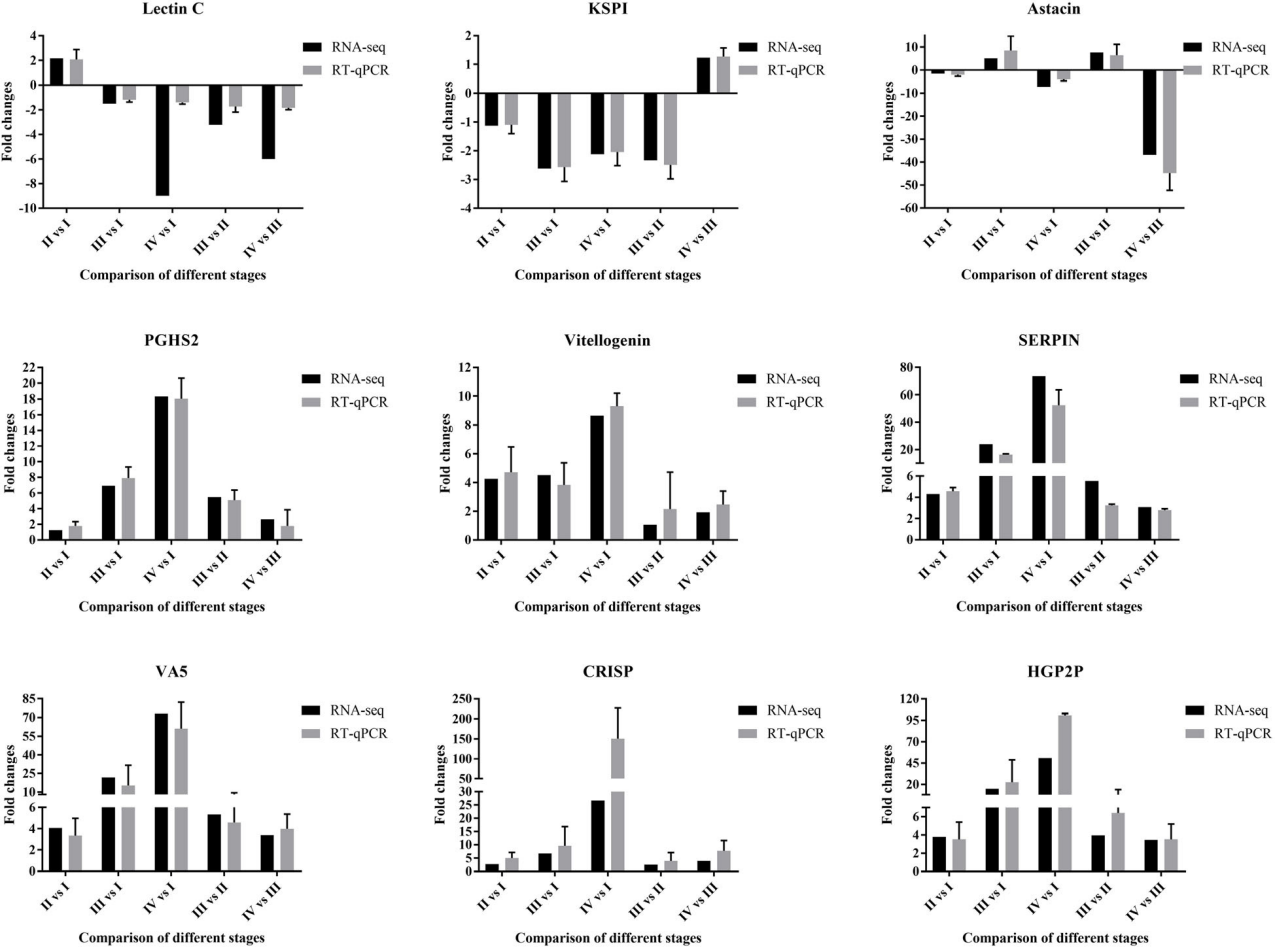
The RT-qPCR validation of DEGs in RNA-seq data. Lectin C: C-type lectin; KSPI: Kazal type serine protease inhibitors; Astacin: a family of multidomain metalloendopeptidases; PGHS2: Prostaglandin G/H synthase 2; Vitellogenin: a protein involved in lipid transport; SERPIN: Serine proteinase inhibitors; VA5: Venom allergen 5; CRISP: Cysteine-rich secretory proteins, Allergen or PR-1-like; HGP2P: hyperglycemic peptide 2 precursor

In order to verify the accuracy of the RNA-seq data in this study, RT-qPCR was conducted to determine the expression levels of the nine genes C-type lectin (Lectin C, TRINITY_DN50699_c3_g1), Kazal type serine protease inhibitors (KSPI, TRINITY_DN50830_c1_g3), Astacin (TRINITY_DN42990_c1_g1), Prostaglandin G/H synthase 2 (PGHS2, TRINITY_DN42030_c3_g1), Vitellogenin (TRINITY_DN52153_c0_g3), Serine proteinase inhibitors (SERPIN, TRINITY_DN43168_c6_g1), Venom allergen 5 (VA5, TRINITY_DN38550_c1_g2), Cysteine-rich secretory proteins (CRISP, TRINITY_DN43203_c3_g2), and hyperglycemic peptide 2 precursor (HGP2P, TRINITY_DN49383_c1_g2), using the ovaries from stage I to stage IV of *P. clarkii*. Three DEGs (VA5, CRISP, and HGP2P) that were up-regulated in all five comparisons, were purposefully selected; the remaining six DEGs (Lectin C, KSPI, Astacin, PGHS2, Vitellogenin, and SERPIN) were randomly selected from the DEGs dataset, three genes were down-regulated and three genes were up-regulated. GAPDH (TRINITY_DN47812_c1_g3) was used as internal reference gene for normalization, because GAPDH was stably expressed in ovary tissues of *P. clarkii* as shown in the RNA-seq data (Supplementary Table S14). The results showed that the change trends of these nine genes detected by RT-qPCR were consistent with those from the RNA-seq data in all five comparison groups (Fig. 10), confirming that the RNA-seq data were authentic.

## Discussion

The red swamp crayfish *Procambarus clarkii* is one of the most well-known invasive species, and it has become an economically important aquatic resource [9]. The delicious taste and high nutritive value are the major reasons for gaining in popularity in China. At present, increasing numbers of farmer are raising *P. clarkii*, and the crayfish-rice culture is the main farming pattern [17]. The production level is continuously increasing, thereby improving the income of farmers. The ovarian development of female *P. clarkii* is an important physiological process representing the reproductive capacity. However, the integrated reproductive pattern of *P. clarkii* remains largely unknown, particularly the molecular mechanisms of ovarian development. This lack of knowledge hinders the production of *P. clarkii* to a large extent. In order to obtain information concerning the molecular mechanisms of ovarian development, we captured the transcriptomics data of four ovarian developmental stages of *P. clarkii* using NGS and compared the difference between different stages in order to understand the developmental pattern.

The ovary of *P. clarkii* consists of oocytes and follicle cells surrounded by a membrane composed of connective tissue and epithelium. The previous studies reported that the ovarian maturation of crayfish proceeds in several stages that can be identified based on morphology and coloration, exhibiting an increase in the size of the ovary as the oocytes proliferate and display uptake of yolk and lipovitellin. The ovarian color changes from light to dark in the order of transparent, translucent white, opaque ivory, yellow, and dark brown [42, 43]. In this study, except for the non-developed ovaries that were transparent, we observed the same color changes of ovaries, and the ovary size continuously increased during the change from white to brown status. We classified the pattern into four stages, the previtellogenic stage (stage I, white), the early vitellogenic stage (stage II, yellow), the middle vitellogenic stage (stage III, light brown), and the mature stage (stage IV, dark brown). Kulkarni et al. (1991) classified the oocyte development of *P. clarkii* into seven stages, and the size of the oocyte increased along with development, including oogonial (< 10 μm), immature (10–65 μm), avitellogenic (66–160 μm), early vitellogenic (161–245 μm), midvitellogenic (246–455 μm), late vitellogenic (456–980 μm), and postvitellogenic-resorptive stages [41]. In the present study, we also found that the size of oocytes of *P. clarkii* increased along with the ovarian development from stage I to stage IV. The sizes of oocytes at stage I in this study was 100–200 μm, corresponding to the avitellogenic stage; however, the sizes at later stages were larger than those at the corresponding stages reported by Kulkarni et al. (1991) [41]. The sizes of middle vitellogenic stage III and mature stage IV in our study were 500–1000 μm and > 1000 μm, respectively. The reasons for the discrepancy might be related to the samples being collected from different areas and at different times. Moreover, the sizes of most oocytes in the same ovary were generally uniform, but some smaller or immature oocytes could also be found in a nearly mature ovary [41]. We also found some immature oocytes in mature ovaries as in previous results, indicating that the oocyte development was asynchronous.

In this study, we performed the transcriptomics of twelve ovaries at four stages, and the total clean read counts of the twelve samples ranged from 42,013,648 to 62,220,956. The total number of bases ranged from 6,118,532,265 bp to 9,054,802,655 bp. The Q30 percentage (94.52–95.58%) of our result was slightly higher than the previous result (91.69%) for mature ovaries of *P. clarkii* [49], demonstrating that our sequencing data were more accurate. The average GC base ratio was 46.99% in our study, which was lower than the result reported by Kang et al. (2019) [49]. The number of samples in our experiment and the Q30 percentage were greater than in the previous assays, and thus we speculated that the GC ratio of our result was more reliable. Jiang et al. (2014) used 454 pyrosequencing to obtain 10,748 isotigs with an N50 of 1794 bp in the ovary library [39]; simultaneously, Shen et al. (2014) used Illumina HiSeq 2000 instrumentation to capture an increasing number of 50,219 non-redundant genes [37]. However, five years later, Kang et al. (2019) used Illumina HiSeq 4000 instrumentation to acquire a total of 105, 957 transcripts (> 200 bp) with an N50 of 1862 bp and 69,261 unigenes with an N50 of 1129 bp [49], far exceeding the results reported by Jiang et al. (2014) [39] and Shen et al. (2014) [37]. In this study, we employed an Illumina HiSeq 2500 to obtain 445,326 transcripts and 216,444 unigenes, of which, 26,934 unigenes comprised more than 1000 bp, more than the previous results mentioned above. Although the N50 values of transcripts and unigenes were 1858 bp and 912 bp, respectively, most of the unigenes (87.56%) were distributed between 200 bp and 1000 bp, similar to the result of Kang et al. (2019) [49]. These data demonstrate that the sequencing information from the Illumina HiSeq 2500 was of high quality and had more content for analysis of the characteristics of the *P. clarkii* ovary. In the transcriptomic research between testis and ovary of *P. clarkii*, 15,667 (69.16%), 13,818 (61.00%), 12,419 (54.83%), 7243 (31.98%), and 6902 (30.47%) genes within the 22,652 annotated genes could be matched with known genes in the NR, Swiss-Prot, KEGG, COG, and GO databases, respectively [39]. Recently, the transcriptomic data of the gills of *P. clarkii* produced 13,948 (40.5%), 5201 (15.1%), 10,747 (31.2%), 13,084 (37.99%), 6381(18.52%), and 13,084 (37.99%) unigenes annotated in NR, NT, Swiss-Prot, PFAM, KOG, and GO databases, respectively [32]. In our transcriptomic data, there were similar numbers of annotated genes in all databases, but due to the high number of unigenes in our results, the annotated proportion was lower than in other reports. However, the number of unigenes annotated in at least one database was higher than in the other reports mentioned above. The best matched species in our data were *Zootermopsis nevadensis*, *Hydra vulgaris*, *Limulus polyphemus*, and *Daphnia magna*, different from the *Daphnia pulex*, *Tribolium castaneum*, *Pediculus humanus corporis*, and *Nasonia vitripennis* reported by Jiang et al. (2014) [39] and *Daphnia pulex*, *Tribolium castaneum*, and *Pediculus humanus* reported by Shen et al. (2014) [37]. The main similarity was that most of these species belong to the Arthropoda.

The correlation analysis of the twelve ovary samples based on the RNA-seq data was analyzed in our study, the three samples in the same stage had high Pearson coefficients (> 0.6), demonstrating that the different samples in the same group were highly correlated. The Pearson coefficients indicated that stage IV was far removed from other stages, and stage II was similar to stage III. In addition, the PCA also showed that each stage could be distinguished from the other stages, and the R value of the Anosim was 0.5926 and highly significant (*p* < 0.001). These data demonstrated that the grouping of the four developmental stages in this study was reliable. The DEG analysis could help to identify the differences in gene expressions in different samples, thereby confirming the relationship between genotype and phenotype. Shui et al. (2012) identified 13 down-regulated and 9 up-regulated proteins of the vitellogenic ovary compared with the previtellogenic ovary of *P. clarkii* through two-dimensional gel electrophoresis, and this result could be helpful for understanding the proteins involved in ovarian development [48]; however, the number of DEGs identified was small. In this study, we found a greater number of DEGs between early vitellogenic ovaries (stage II) and previtellogenic ovaries (stage I) by NGS technology, demonstrating that the results from this study could provide more information for analyzing ovarian development. Furthermore, among the four different stages, we found that the difference between the mature ovary (stage IV) and the previtellogenic ovary (stage I) was greater than in other comparisons, the second largest difference was between the mature ovary (stage IV) and the middle vitellogenic ovary (stage III), while the difference between the middle vitellogenic ovary (stage III) and the early vitellogenic ovary (stage II) was less than in other comparison groups. These findings demonstrated that the mature stage was the most important for ovulation through major changes involving many genes.

GO classification can be utilized to analyze the functional categorization of unigenes or DEGs in transcriptomic analysis of most species, including crustaceans. For *Portunus trituberculatus*, the molecular function (the binding and catalytic activity constituted the majority of the categories) was the most assigned, followed by biological process (the cellular process and metabolic process were the predominant terms) and cellular components (the cell, cell part, and organelle were the major categories in cellular components) [68]. In black tiger shrimp, the biological process was the highest annotated, followed by cellular component and molecular function, this was different from the *Portunus trituberculatus* results, while the subgroups in these three categories were the same as for *Portunus trituberculatus* [69]. Also, as a crustacean, the previous ovarian transcriptomic analysis of *P. clarkii* [37, 39, 49] revealed that the GO annotation was similar to those of *Portunus trituberculatus* and the black tiger shrimp; as expected, we also got the similar results in the GO annotation in this study. The DEGs between different stages indicated that the differences among stages I, II and III were relatively low, while the difference between stages IV and III was more obvious than in other comparison groups of two adjacent stages. Notably, the GO term of nutrient reservoir activity within molecular function was significant at stages II and III compared with stage I, indicating that these stages provide enough nutrient for subsequent ovarian development. In addition, the GO terms of cell (GO:0005623), intracellular (GO:0005622), and organelle (GO:0043226) were significantly prominent between stages IV and III, speculating that these GO terms played important roles in ovarian mature. Furthermore, to understand the complex biological processes of genes, the KEGG database was used to analyze functional information of metabolic pathways or regulatory networks of genes in cells. In this study, 3560 unigenes were annotated in the KEGG database; the subgroups of signal transduction, endocrine system, transport and catabolism, and translation were the most prominent, similar to the result of Meng et al. (2015) [68]. We found that the PI3-Akt signaling pathway (ko04151), endocytosis (ko04144), purine metabolism (ko00230), and RNA transport (ko03013) were the distinct pathways. Simultaneously, the PI3K-Akt signaling pathway, the Wnt signaling pathway, the GnRH signaling pathway, progesterone-mediated oocyte maturation, the insulin signaling pathway and the TGF-beta signaling pathway were identified, and these were related to gonadal development and maturation and were assigned to the subgroups of signal transduction and endocrine system as in previous studies [68, 69]. In mammals, the PI3K-Akt signaling pathway is indispensable for the regulation of cell proliferation, survival, migration, and metabolism in different tissues, and it plays an essential role in oocyte growth and the activation of primordial follicles in the ovary [70–73]. Interestingly, we found that the PI3K-Akt signaling pathway was only significant at stage II compared with stage I, indicating that this pathway played a vital role for the activation of primordial follicles and oocyte growth in early vitellogenic ovaries of *P. clarkii*. Furthermore, various “nutrient sensing” mechanisms might take part in forming the link between nutrient status and folliculogenesis, while the PI3K-Akt pathway could sense nutrient flux from within the follicle [74]. Amazingly, the GO term of nutrient reservoir activity was significant at stage II compared with stage I, corresponding to the PI3K-Akt pathway. Therefore, we speculated that the PI3K-Akt pathway could sense the nutrient reservoir activity to regulate oocyte growth and activation of primordial follicles in early vitellogenic ovaries of *P. clarkii*. Finally, apoptosis, lysine biosynthesis, and the NF-kappa B signaling pathway were significant at stage III compared with stage II, indicating that stage III was related to immune response and biosynthesis; cell cycle and the p53 signaling pathway were prominent at stage IV compared with stage III, showing that stage IV was involved with cell proliferation and DNA replication, corresponding to the GO term of cell cycle regulation reported by Jiang et al. (2014) [39]. These data demonstrated that different stages have different pathways for regulating the ovarian development of *P. clarkii*.

## Conclusion

In summary, we compared the transcriptomics among four ovarian stages of *P. clarkii* through Illumina sequencing technology, including the previtellogenic stage, the early vitellogenic stage, the middle vitellogenic stage, and the mature stage. We found that the expression patterns of the first three stages defined in this study were similar, but each stage also had its own uniquely expressed modules that distinguished it from other stages. The expression pattern of the mature stage was quite different from those of the other three stages. The GO term of nutrient reservoir activity played a foreshadowing role at the early vitellogenic stage, and the terms of cell, intracellular, and organelle were prominent at the mature stage. The KEGG analysis revealed that the early ovarian development of *P. clarkii* was mainly associated with the PI3K-Akt signaling pathway and focal adhesion, while the middle ovarian development was related to apoptosis, lysine biosynthesis, and the NF-kappa B signaling pathway, and the late ovarian development was involved with the cell cycle and p53 signaling pathway. These data provide insights into the molecular mechanisms of ovarian development, and may help to improve the reproductive performance of *P. clarkii*.

## Supplementary Information

**Additional file 1: Supplementary Figure S1**: The base content of twelve ovary samples. **Supplementary Figure S2**: The information of the assembly transcripts of the *P. clarkii* ovary. **Supplementary Figure S3**: The repeatable correlation of ovary samples at the same stage of *P. clarkii*. **Supplementary Figure S4**: The correlation of the different stages of *P. clarkii* ovary. **Supplementary Figure S5**: The TPM analysis of the different stages of *P. clarkii* ovary. **Supplementary Figure S6**: The gene expression patterns within irregular subclusters of DEGs of twelve ovary samples. **Supplementary Figure S7**: The GO classification of DEGs in different comparison groups. **Supplementary Figure S8**: The KEGG classification of DEGs in different comparison groups. **Supplementary Table S1**: Primers used for RT-qPCR.

**Additional file 2.**

**Additional file 3.**

**Additional file 4.**

**Additional file 5.**

**Additional file 6.**

**Additional file 7.**

**Additional file 8.**

**Additional file 9.**

**Additional file 10.**

**Additional file 11.**

**Additional file 12.**

**Additional file 13.**

**Additional file 14.**

## Data Availability

All data generated or analyzed during this study are available in this article and its supplementary information files. The transcriptomic raw data in the current study have been deposited to the NCBI SRA database with the accession number of PRJNA690634 (https://www.ncbi.nlm.nih.gov/sra/PRJNA690634).
