# Supplementary material for: Comparative transcriptomic analysis of the different developmental stages of ovary in red swamp crayfish Procambarus clarkii
Source: BMC Genomics. 2021 Mar 21;22:199. doi: 10.1186/s12864-021-07537-x (PMC7981885; doi:10.1186/s12864-021-07537-x)
Supplement: Supplementary file 1 — Additional file 1: Supplementary Figure S1: The base content of twelve ovary samples. Supplementary Figure S2: The information of the assembly transcripts of the P. clarkii ovary. Supplementary Figure S3: The repeatable correlation of ovary samples at the same stage of P. clarkii. Supplementary Figure S4: The correlation of the different stages of P. clarkii ovary. Supplementary Figure S5: The TPM analysis of the different stages of P. clarkii ovary. Supplementary Figure S6: The gene expression patterns within irregular subclusters of DEGs of twelve ovary samples. Supplementary Figure S7: The GO classification of DEGs in different comparison groups. Supplementary Figure S8: The KEGG classification of DEGs in different comparison groups. Supplementary Table S1: Primers used for RT-qPCR. [file 12864_2021_7537_MOESM1_ESM.docx]

**Comparative transcriptomic analysis of** **the different developmental stages of ovary in red swamp crayfish *Procambarus clarkii***

Yizhi Zhong^1^, Wenbin Zhao^1^, Zhangsheng Tang^1^, Liming Huang^1^, Xiangxing Zhu^2^, Xiang Liang^3^, Aifen Yan^2^, Zhifa Lu^1^, Yanling Yu^1^, Dongsheng Tang^2^, Dapeng Wang^1,*^ and Zhuanling Lu^1,^^*^

^1^Guangxi Academy of Fishery Sciences/Guangxi Key Laboratory of Aquatic Genetic Breeding and Healthy Aquaculture, Nanning 530021, China;

^2^Guangdong Provincial Key Laboratory of Animal Molecular Design and Precise Breeding, Guangdong Provincial Engineering and Technology Research Center for Gene Editing, School of Medical Engineering, Foshan University, Foshan 528225, China;

^3^Development Research Institute of Agro-animal Husbandry Industry, Guangxi University, Nanning 530004, China.

*Corresponding authors, E-mail: nicky.004@163.com (ZL Lu); oucwdp@163.com (DP Wang)

**Table of Contents**

Supplementary Figure S1: The base content of twelve ovary samples.

Supplementary Figure S2: The information of the assembly transcripts of the *P. clarkii* ovary.

Supplementary Figure S3: The repeatable correlation of ovary samples at the same stage of *P. clarkii*.

Supplementary Figure S4: The correlation of the different stages of *P. clarkii* ovary.

Supplementary Figure S5: The TPM analysis of the different stages of *P. clarkii* ovary.

Supplementary Figure S6: The gene expression patterns within irregular subclusters of DEGs of twelve ovary samples.

Supplementary Figure S7: The GO classification of DEGs in different comparison groups.

Supplementary Figure S8: The KEGG classification of DEGs in different comparison groups.

Supplementary Table S1: Primers used for RT-qPCR.


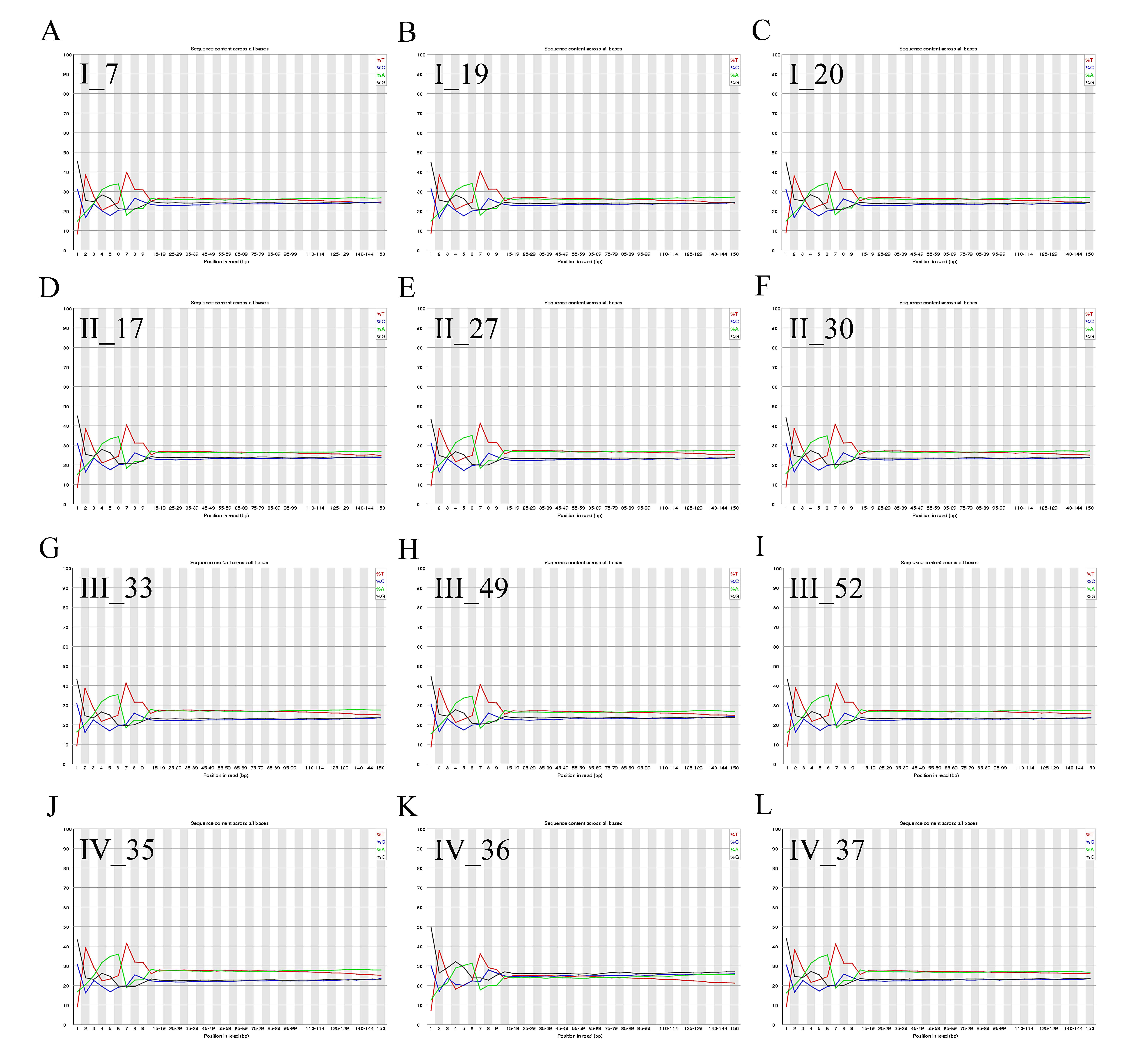


**Supplementary Figure S1** The base content of twelve ovary samples. A-C: Ovaries at stage I, D-F: Ovaries at stage II, G-I: Ovaries at stage III, J-L: Ovaries at stage IV. Horizontal axis represents the base site of reads, vertical axis represents the percentage of the four bases.


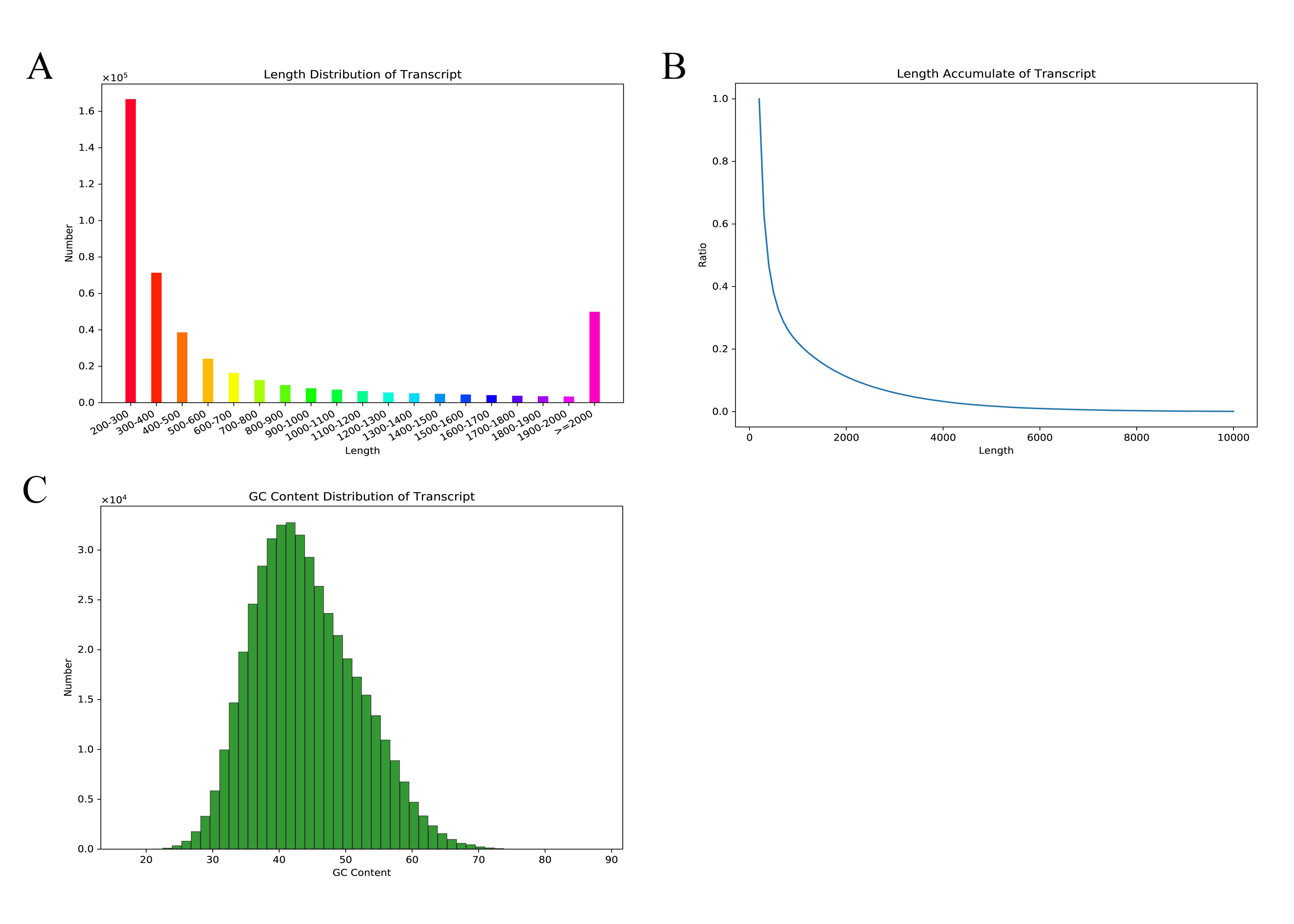


**Supplementary Figure S2** The information of the assembly transcripts of the *P. clarkii* ovary. A: The length distribution of transcripts after assembly, the abscissa represents the length range of the transcripts, the ordinate represents the number of transcripts corresponding to the length. B: The length accumulate of transcripts after assembly, the abscissa represents the length of transcript, the ordinate represents the ratio of transcripts more than the corresponding length. C: The GC content distribution and the corresponding numbers of transcripts.


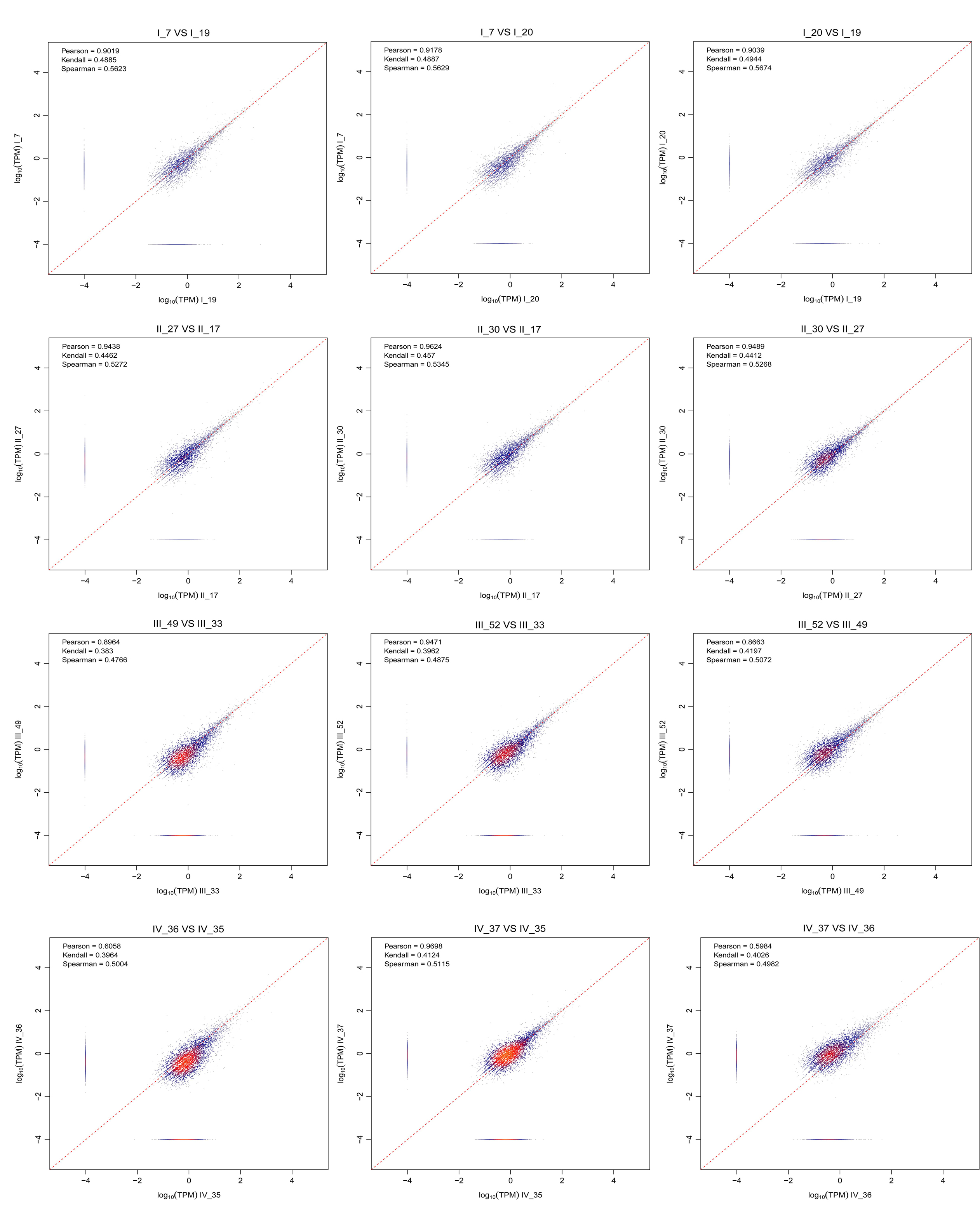


**Supplementary Figure S3** The repeatable correlation of ovary samples at the same stage of *P. clarkii*. Pearson, Kendall and Spearman correlation coefficient are showed in the pictures. The more similar the samples, the closer the correlation index is to 1, and most of the points are around the diagonal.


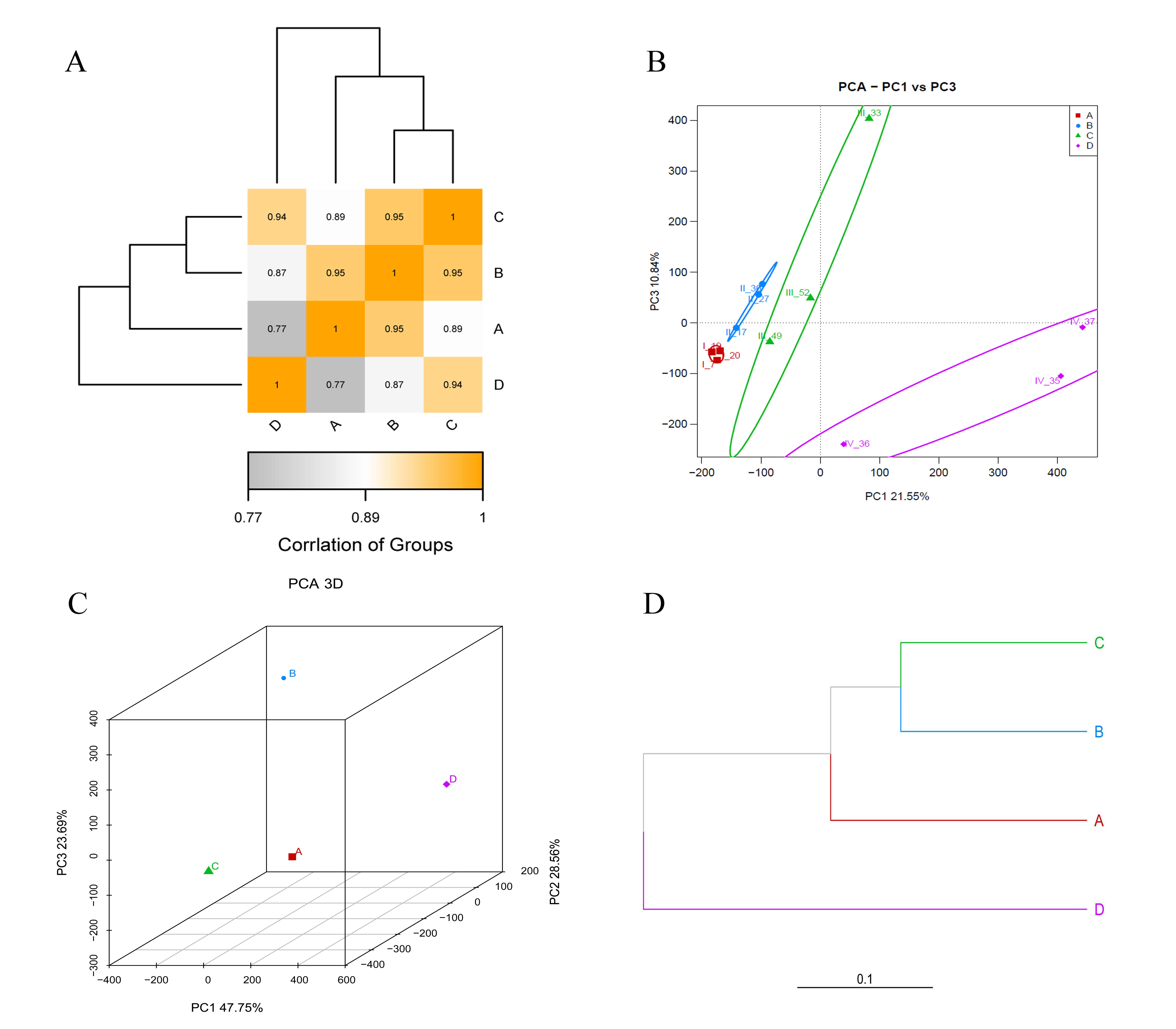


**Supplementary Figure S4** The correlation of the different stages of *P. clarkii* ovary. A: The heatmap of the Pearson coefficients among the four stages. The more grey the color of the block, the lower correlation between two stages; the more yellow the color, the higher correlation between two stages. B: The Principal Component Analysis (PCA) of the twelve ovary samples by comparing the PC1 with PC3. C: The PCA of the four groups of stages. D: Hierarchical clustering based on the distances among the four groups of stages. The length of the branch represents the distance between the different stages, the more similar the samples, the closer they get. Note: The alphabet of A, B, C and D in the graph represent the stage I, stage II, stage III and stage IV, respectively.


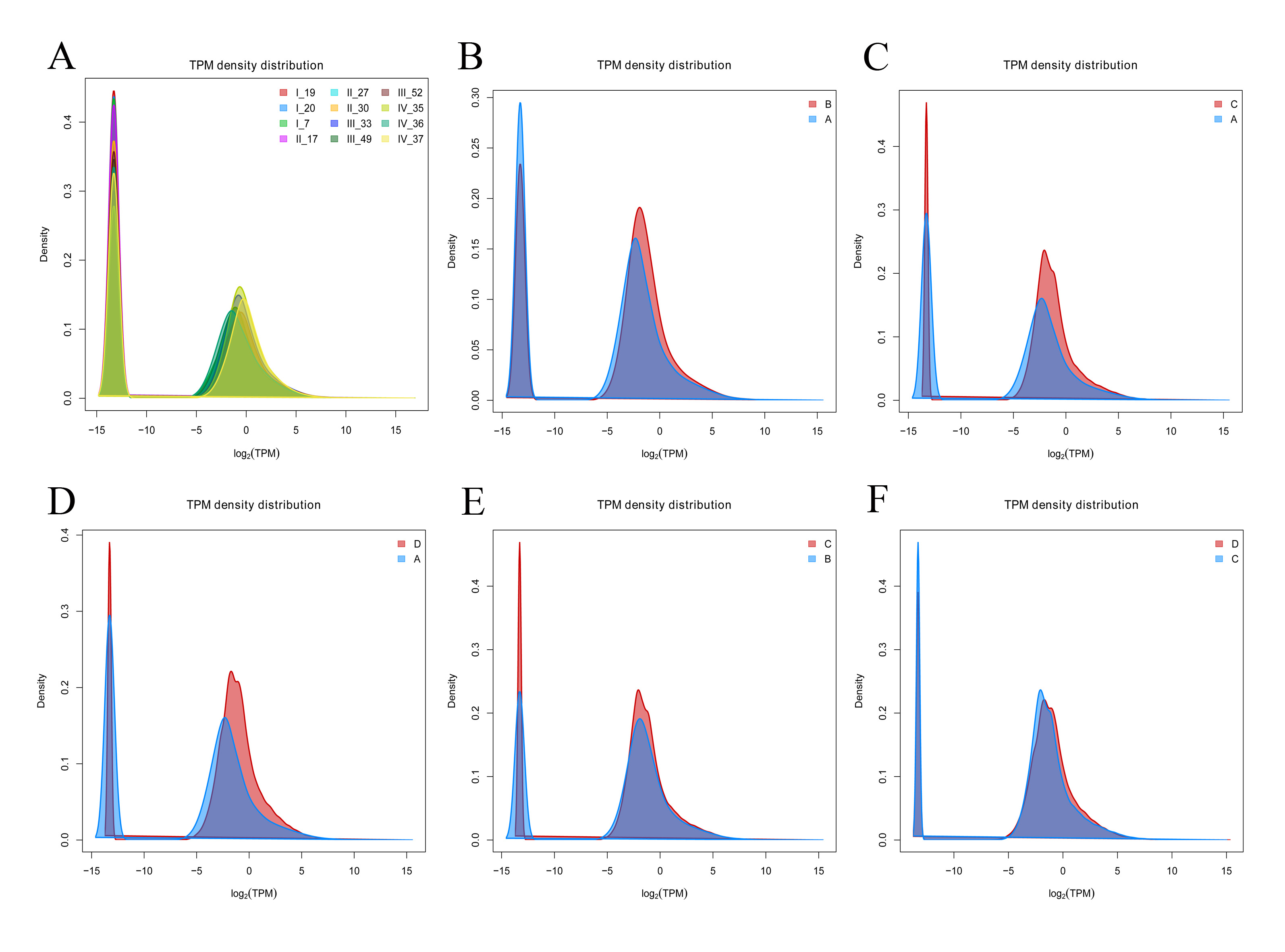


**Supplementary Figure S5** The TPM analysis of the different stages of *P. clarkii* ovary. A: The TPM density distribution of twelve ovary samples. B: The TPM density distribution between stage II and stage I. C: The TPM density distribution between stage III and stage I. D: The TPM density distribution between stage IV and stage I. E: The TPM density distribution between stage III and stage II. F: The TPM density distribution between stage IV and stage III. The abscissa is the value of log_2_(TPM), the higher the value, the higher the gene expression. The ordinate is the corresponding relative density value. The area of the density curve of each sample is 1.


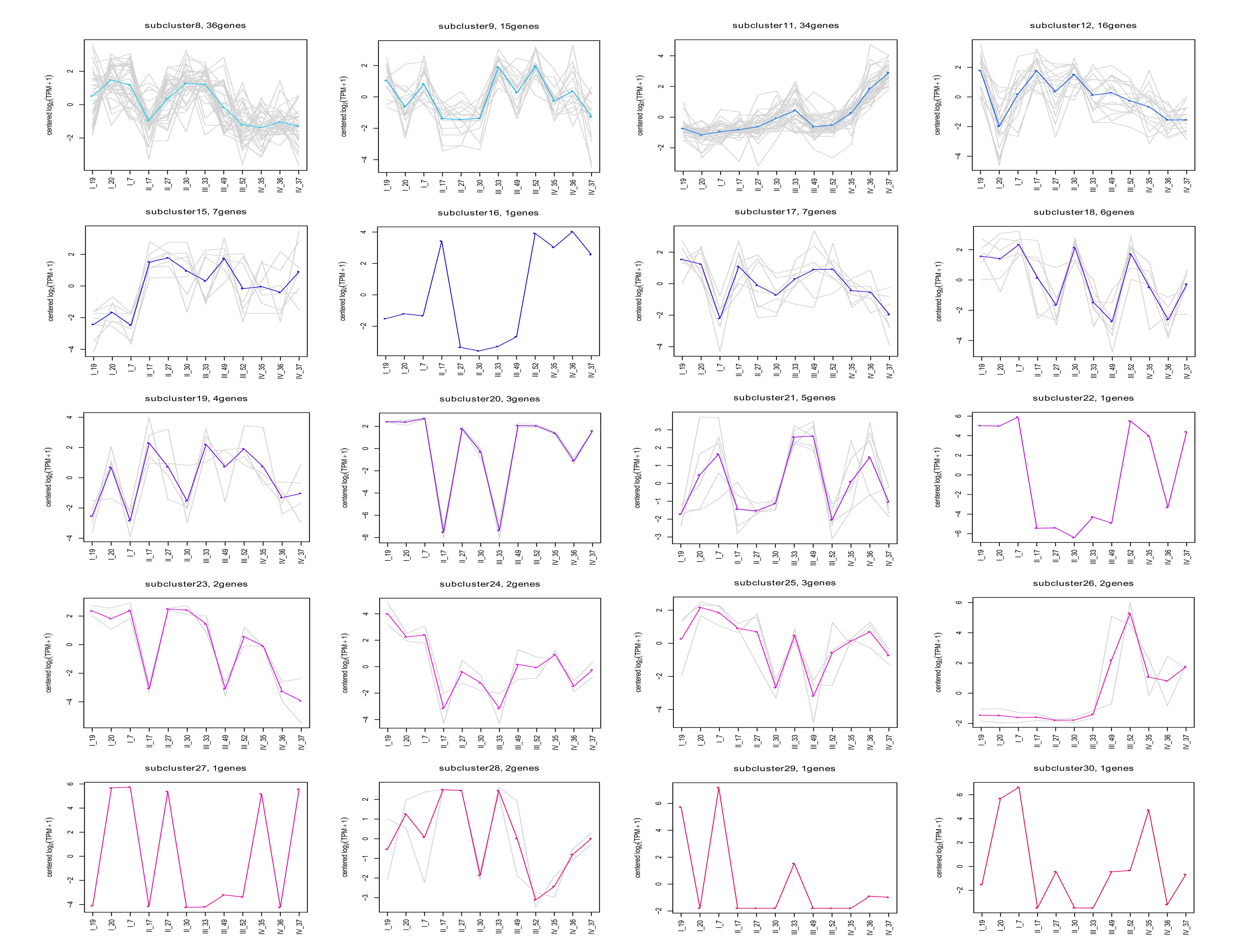


**Supplementary Figure S6** The gene expression patterns within irregular subclusters of DEGs of twelve ovary samples.


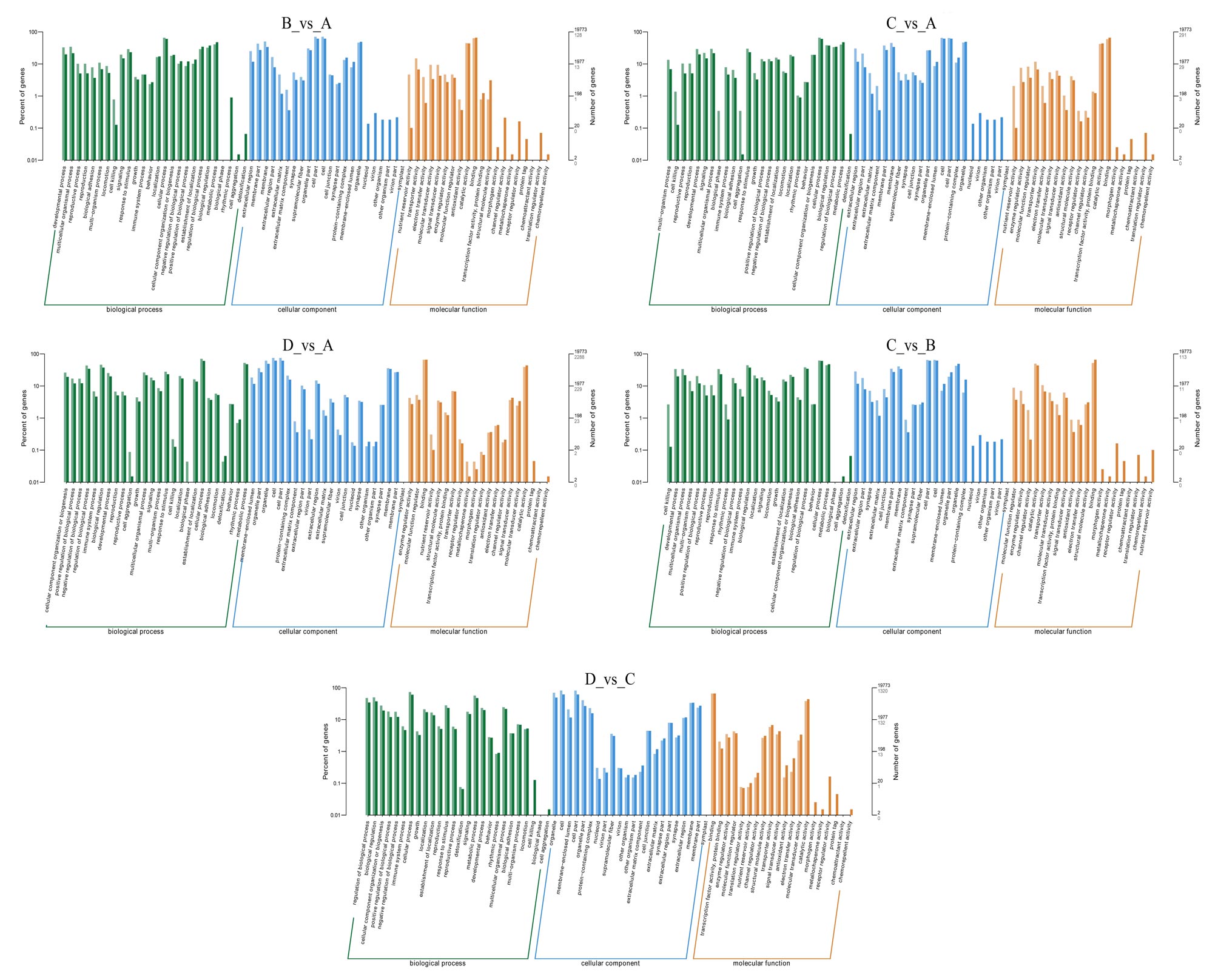


**Supplementary Figure S7** The GO classification of DEGs in different comparison groups. B_vs_A is stage II_vs_stage I. C_vs_A is stage III_vs_stage I. D_vs_A is stage IV_vs_stage I. C_vs_B is stage III_vs_stage II. D_vs_C is stage IV_vs_stage III.


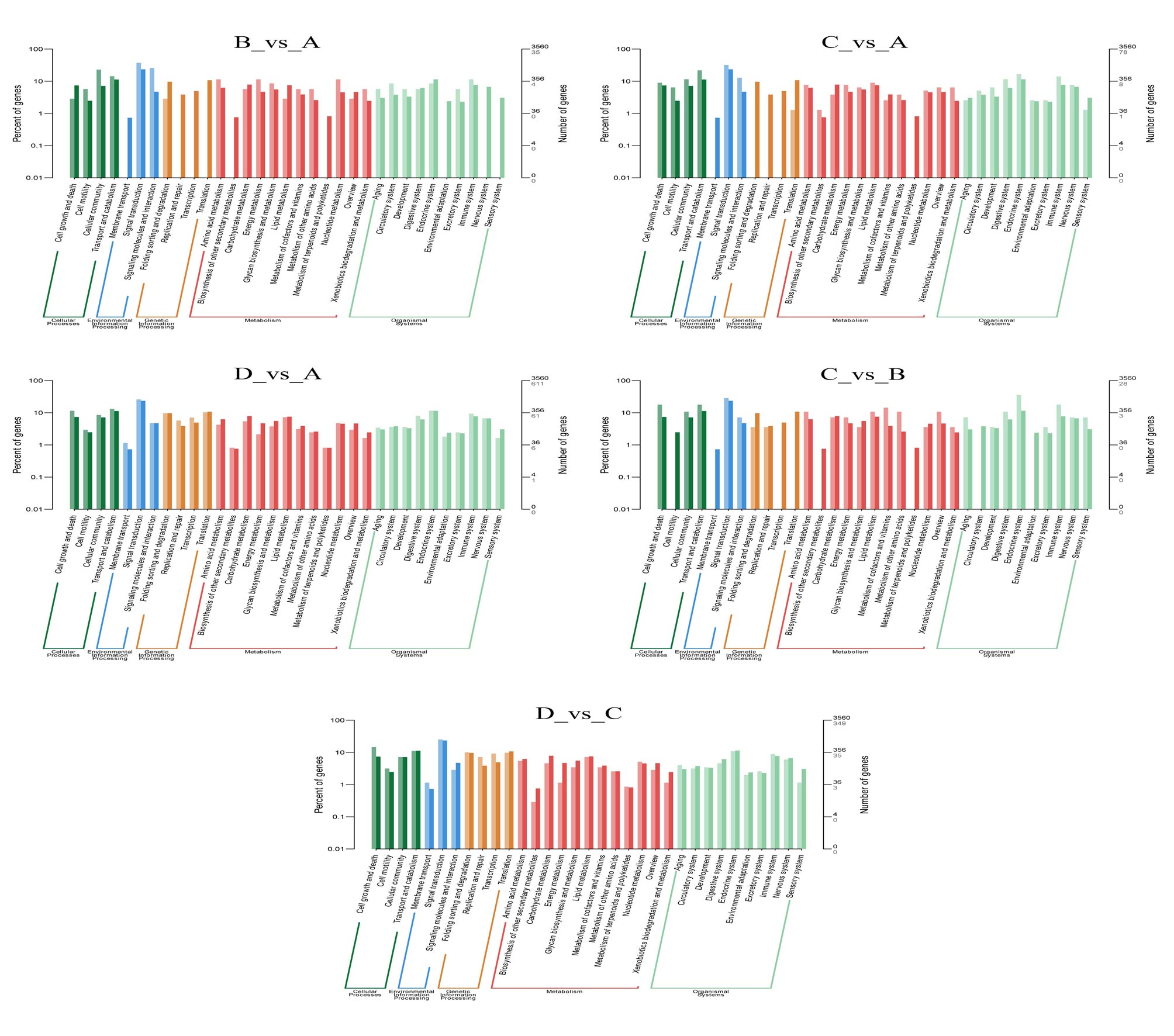


**Supplementary Figure S8** The KEGG classification of DEGs in different comparison groups. B_vs_A is stage II_vs_stage I. C_vs_A is stage III_vs_stage I. D_vs_A is stage IV_vs_stage I. C_vs_B is stage III_vs_stage II. D_vs_C is stage IV_vs_stage III.

Supplementary Table S1 Primers used for RT-qPCR

| Gene ID | Gene name | Primer name | Sequence (5’-3’) | Product length (bp) |
| --- | --- | --- | --- | --- |
| TRINITY_DN50699_c3_g1 | C-type lectin | Lectin C-F | AGTGTGCTGGCAGACACATC | 148 |
|  |  | Lectin C-R | CCTTCCGGCATAACATCATC |  |
| TRINITY_DN50830_c1_g3 | Kazal type serine protease inhibitors | KSPI-F | CCAGGGTCCAGGTCAGTAAA | 120 |
|  |  | KSPI-R | AATCAGCGGGTCAAAGGTC |  |
| TRINITY_DN42990_c1_g1 | Astacin | Astacin-F | AGCGACTTTACACGCCTTCA | 189 |
|  |  | Astacin-R | GACTACTCATCGGACATGCACTAC |  |
| TRINITY_DN42030_c3_g1 | Prostaglandin G/H synthase 2 | PGHS2-F | CTACTGCATCAATGTCCACG | 111 |
|  |  | PGHS2-F | CCTACAGGAAGAGGTTTGAG |  |
| TRINITY_DN52153_c0_g3 | Vitellogenin | Vg-F | ATCTACTCCAGCATCACCTG | 118 |
|  |  | Vg-R | GGTCGTTGGATTCTGAGATG |  |
| TRINITY_DN48366_c0_g1 | Serine proteinase inhibitors | SERPIN-F | AAGAAGCAGGCACTGGAGG | 212 |
|  |  | SERPIN-R | CTGTCGGTCATGTCAAAGCA |  |
| TRINITY_DN38550_c1_g2 | Venom allergen 5 | VA5-F | CATACTGTGGTTGTGGTGGA | 122 |
|  |  | VA5-R | ACATCTTACCTGAGAGCCAG |  |
| TRINITY_DN43203_c3_g2 | CRISP | CRISP-F | TCACTGGTTCCTCCTCATTG | 122 |
|  |  | CRISP-R | TCATCGTACCAACCATGCAC |  |
| TRINITY_DN49383_c1_g2 | hyperglycemic peptide 2 precursor | HGP2P-F | ATTCATACGCATTCAGAACAGC | 138 |
|  |  | HGP2P-R | GACATTACCAAGAGGGAGAGGA |  |
|  | GAPDH | GAPDH-F | GCCCAGAACATCATCCCATCT | 235 |
|  |  | GAPDH-R | CGTCATCCTCAGTGTAACCCAAG |  |
